# The pH Signaling Transcription Factor PAC-3 Regulates Metabolic and Developmental Processes in Pathogenic Fungi

**DOI:** 10.3389/fmicb.2019.02076

**Published:** 2019-09-04

**Authors:** Maíra Pompeu Martins, Nilce M. Martinez-Rossi, Pablo R. Sanches, Eriston Vieira Gomes, Maria Célia Bertolini, Wellington R. Pedersoli, Roberto Nascimento Silva, Antonio Rossi

**Affiliations:** ^1^Department of Genetics, Ribeirão Preto Medical School, University of São Paulo, Ribeirão Preto, Brazil; ^2^Department of Biofunctional, Morgana Potrich College, Mineiros, Brazil; ^3^Department of Biochemistry and Technological Chemistry, São Paulo State University, UNESP, Institute of Chemistry, Araraquara, Brazil; ^4^Department of Biochemistry and Immunology, Ribeirão Preto Medical School, University of São Paulo, Ribeirão Preto, Brazil

**Keywords:** *Neurospora crassa*, mycorrhizal association, phytopathogen, inorganic orthophosphate, RNA-sequencing

## Abstract

The zinc finger transcription factor PAC-3/RIM101/PacC has a defined role in the secretion of enzymes and proteins in response to ambient pH, and also contributes to the virulence of species. Herein we evaluated the role of PAC-3 in the regulation of *Neurospora crassa* genes, in a model that examined the plant-fungi interactions. *N. crassa* is a model fungal species capable of exhibiting dynamic responses to its environment by employing endophytic or phytopathogenic behavior according to a given circumstance. Since plant growth and productivity are highly affected by pH and phosphorus (P) acquisition, we sought to verify the impact that induction of a Δ*pac-3* mutation would have under limited and sufficient Pi availability, while ensuring that the targeted physiological adjustments mimicked ambient pH and nutritional conditions required for efficient fungal growth and development. Our results suggest direct regulatory functions for PAC-3 in cell wall biosynthesis, homeostasis, oxidation-reduction processes, hydrolase activity, transmembrane transport, and modulation of genes associated with fungal virulence. Pi-dependent modulation was observed mainly in genes encoding for transporter proteins or related to cell wall development, thereby advancing the current understanding regarding colonization and adaptation processes in response to challenging environments. We have also provided comprehensive evidence that suggests a role for PAC-3 as a global regulator in plant pathogenic fungi, thus presenting results that have the potential to be applied to various types of microbes, with diverse survival mechanisms.

## Introduction

Soil nutrient cycling is primarily performed by microorganisms and serves to support plant development through the uptake of soil minerals and by facilitating the decomposition of organic matter. Phosphorus (P) acquisition and soil pH are two of the most influential factors affecting plant growth and productivity and are regulated primarily by the indigenous microbial community. Moreover, studies have suggested that changes in the composition of the soil fungal community are strongly correlated with changes in soil pH, which subsequently controls the availability of carbon (C), nitrogen (N) and phosphorus (P) (Hinsinger, 2001; Kemmitt et al., 2006; Rousk et al., 2009; Zhang et al., 2016).

In the soil, P exists as inorganic orthophosphate (Pi), either as H_2_PO_4_^–^ or HPO_4_^2–^, depending on the soil pH, or less commonly, as organic Pi (phytates). Uptake of P occurs via two distinct routes. The first is direct and occurs through plant roots. The second route is indirect and occurs via mycorrhizal symbiosis. Briefly, the host plant obtains P primarily from soil fungal partners supported by chemotropism, which is the ability of fungi to sense and grow toward a chemical gradient. The association with specialized soil fungi, and subsequent formation of mycorrhizal roots, serves to improve and increase the P content in plants. However, the beneficial effect afforded by the development of mycorrhizal symbiosis varies according to the fungal species involved (Hinsinger, 2001; Plassard et al., 2011; Johri et al., 2015; Turrà and Di Pietro, 2015).

Despite the importance of fungus-plant interactions, our current knowledge regarding the molecular regulation of these systems is rudimentary. However, recent advances have been made in the genomics of fungal model systems, which have contributed to our understanding of the cellular machinery responsible for regulating the chemoattraction of filamentous hyphae by plant roots, thereby enabling plants to forage for essential nutrients (Turrà and Di Pietro, 2015).

An example of a model for the study of plant-fungi interactions is the filamentous fungus *Neurospora crassa*, which has contributed significantly to the development of modern genomic studies. *N. crassa* grows on decaying plant material, and is capable of exhibiting endophytic or phytopathogenic behavior depending on the specific environmental circumstances (Kuo et al., 2014). Further, this fungal species has been shown to express defense- and pathogenicity-related genes including oxidoreductases and necrosis-inducing proteins (Leal et al., 2009; Kuo et al., 2014). It has also exhibited the ability to synthesize and secrete cellulases and hemicellulases, which function in the degradation of lignocellulosic material (Kuo et al., 2014; Kourist et al., 2015; Antonieto et al., 2017). Thus, *N. crassa* serves as a model organism for the investigation of a wide range of plant-fungal relationships.

The availability of P and the modulation of soil pH are important limiting factors in plant health and development, and thus, the ability to rapidly adapt to the environment via modulation of specific genes, is critical for efficient utilization of the available nutrients and overall survival. The transcription factor PAC-3, a new designation for *N. crassa* PacC (Virgilio et al., 2016), has a well-defined role in the pH-signaling pathway, and has also been shown to be required for virulence in specific plant pathogens, namely, *Penicillium digitatum* (Zhang et al., 2013), *Fusarium oxysporum* (Caracuel et al., 2003b), *Sclerotinia sclerotiorum* (Rollins, 2003), *Colletotrichum gloeosporioides* (Miyara et al., 2008), and *Colletotrichum acutatum* (You and Chung, 2007), thus suggesting a critical role for this protein in host-pathogen interactions.

Herein we present an overview of the effect induced following deletion of *pac-3* in *N. crassa* when cultivated in media containing low and high concentrations of Pi. Results from the Gene Ontology (GO) enrichment analysis indicated that the most highly modulated gene groups were integral to the membrane, or with associated with oxidation-reduction processes, hydrolase activity, and transmembrane transport.

Our results suggest potential regulatory mechanisms employed by *pac-3* in a ubiquitous filamentous fungus, thereby providing a comprehensive overview of genetic modulation in a model organism that is proficient in deconstruction of plant cell walls (Antonieto et al., 2017). These results, therefore, contribute to the understanding of possible mechanisms associated with plant-pathogen interactions.

## Materials and Methods

### *Neurospora crassa* Strains and Culturing Conditions

The *N. crassa* Δ*pac-3* knockout strain (*pac-3*^KO^) was generated by replacing the *pacC* open reading frame (ORF; NCU00090) with the bar gene from a *mus-52*^KO^ background strain. Gene knockout in the mutant strain was confirmed via polymerase chain reaction (PCR) using specific oligonucleotides (Cupertino et al., 2012). All strains were maintained on solid Vogel’s Minimal (VM) medium supplemented with 2% sucrose at 30°C and pH 5.8 (Vogel, 1956).

Following 5 days (Δ*mus-52*) or 10 days (Δ*pac-3*) of culturing consisting of 3 days at 30°C in the dark and 2 (Δ*mus-52*) or 7 (Δ*pac-3*) days at room temperature in ambient light, conidia (approximately 10^7^ cells/mL^–1^) were germinated in an orbital shaker for 5 h at 30°C (200 rpm) in low- and high-Pi media (final concentrations, 10 μM or 10 mM Pi, respectively). Media was supplemented with 44 mM sucrose as the carbon source, and 50 mM of sodium citrate was used to adjust the pH to 5.4, as previously described (Nyc et al., 1966; Nahas et al., 1982; Gras et al., 2007). The mycelium was then harvested, frozen in liquid nitrogen, and stored at −80°C until RNA isolation was performed. All experiments were performed in three biological replicates.

### RNA Extraction

Total RNA was isolated using TRIzol^TM^ Reagent (Invitrogen, United States) according to manufacturer’s instructions and treated with DNAse I, RNAse-free (Thermo Fisher, United States). The RNA concentration was quantified using a NanoDrop ND-1000 spectrophotometer (Thermo Fisher). RNA integrity was determined via agarose electrophoresis and by employing the Agilent Bioanalyzer platform 2100 (Agilent, United States).

### RNA-Seq Data Analysis

Four cDNA libraries were sequenced (Δ*mus-52* and Δ*pac-3* strains, cultivated in media with high- or low-Pi concentration), with their respective biological triplicates to generate 100 bp paired-end reads using an Illumina HiSeq 2000 sequencer (Illumina, United States). To assess the library quality prior to and after trimming, we used the FastQC software. To remove the sequencing bases from the end of the reads, we applied a minimum Phred score of 20. The genome of *N. crassa*^1^ and the Bowtie2 software (Langmead and Salzberg, 2012) were then employed to map the filtered reads. The coverage and alignments of the transcripts were visually inspected using Integrative Genomics Viewer software (IGV) (Robinson et al., 2011; Thorvaldsdottir et al., 2012). After performing library alignment and quality filtering steps, read count values were obtained. Once the read count table was generated, differential expression and statistical analysis were performed using the DESeq2 Bioconductor package (Love et al., 2014).

We adjusted the *p*-value threshold set to 0.05 with a log_2_ fold change ±1.5 using the Benjamini-Hochberg procedure (Benjamini and Hochberg, 1995) to denote statistical significance in changes for a given gene’s expression levels. Functional annotation was performed according to GO (Blake and Harris, 2008), using the Bast2GO software (Gotz et al., 2008). The BayGO algorithm was then applied to perform GO term enrichment (Vencio et al., 2006).

### Validation of RNA-Seq by RT-qPCR

One microgram of total RNA, extracted from the fungal mycelia, was reverse transcribed into complementary DNA (cDNA) using the High Capacity cDNA Reverse Transcription Kit (Applied Biosystems, United States) according to manufacturer’s instructions. The expression pattern for the selected genes was quantified via real-time quantitative reverse transcription polymerase chain reaction (qRT-PCR) using Power SYBR Green PCR Master Mix (Applied Biosystems) with the StepOnePlus Real-Time PCR System (Applied Biosystems) for 40 cycles. The initial denaturation step was performed at 50°C for 2 min and at 95°C for 10 min, followed by 40 cycles at 95°C for 15 s and 60°C for 1 min. Specific primer sequences used as well as the concentration in each reaction, and reaction efficiencies are presented in Supplementary Table S1. Two reference genes, namely, actin (NCU04173), and β-tubulin (NCU04054) were used as controls. Each gene had its relative expression level quantified using the Livak (2^–ΔΔCt^) method (Livak and Schmittgen, 2001). We performed statistical analysis using Student *t*-tests with GraphPad Prism v. 5.1 software.

### The Analysis of Putative PAC-3 Binding Sites *in silic*o

The complete genome sequence of *N. crassa* OR74A was used to obtain the 5′upstream regions (1 kb) for each gene. The occurrence of the PAC-3 motif, 5′-GCCARG-3′, was determined using an *ad hoc* perl script.

## Results

### The Transcriptional Response of the *N. crassa* Δ*pac*-3-Mutant

**FIGURE 1:**
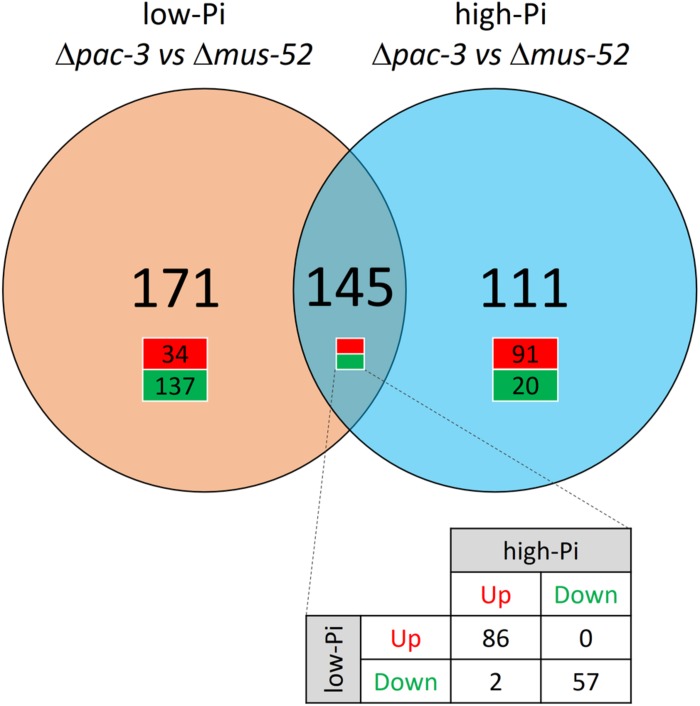
Venn diagram indicating the number of DEGs identified in the mutant Δ*pac-3* strain (test) compared to the control (Δ*mus-52* strain) in media containing low-Pi (orange) and high-Pi (blue). The green color indicates down-regulated genes and the red color indicates up-regulated genes. Thresholds for the classification of DEGs were (*P* < 0.05), up-regulated: log_2_ (Fold Change) ≥1.5 or down-regulated: log_2_ (Fold Change) ≤−1.5.

Results from Bowtie2 analysis revealed that approximately 84–86% of the total high-quality reads were found to align with the reference genome of *N. crassa* (Langmead and Salzberg, 2012; Supplementary Table S2). Furthermore, we identified 145 DEGs that were modulated regardless of the Pi concentration, however, were responsive to deletion of *pac-3*; while 171 DEGs were found to be modulated concomitantly with the deletion of *pac-3* and with low-Pi availability. An additional 111 DEGs were found to be responsive to *pac-3* deletion and high-Pi concentration (Figure 1). For each condition analyzed, a list of all DEGs was generated and is presented in Supplementary Table S3.

**FIGURE 2:**
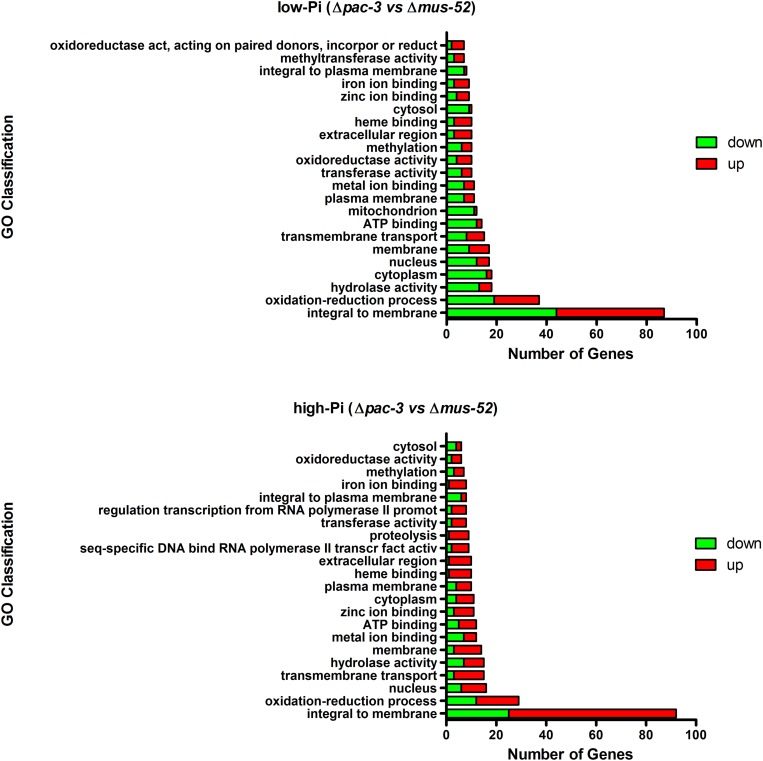
Gene Ontology-based functional categorization of the most representative differentially expressed genes (*P* < 0.05). The red and green bars indicate the number of up- and down-regulated genes, respectively.

An analysis of the relevant biological processes associated with the DEGs was conducted to increase the understanding of the functional categories affected by the deletion of *pac-3.* The most representative gene group in both Pi conditions was identified as being integral to membranes, followed by those that are related to oxidation-reduction processes. Genes related to hydrolase activity, transmembrane transport, cytoplasm, and nucleus were also significantly represented in both analyzed Pi conditions (Figure 2). Further, the most significantly affected DEGs in both nutritional conditions were plotted in a volcano plot, highlighting those genes with log_2_ fold change ≥ or ≤5.0 (Supplementary Figure S1).

### Modulation of Genes Identified as Integral to Membrane and Transmembrane Transport

A large high number of genes were identified as being modulated in response to both high- and low-Pi concentrations, thereby revealing a transcriptional response that was exclusive to the deletion of *pac-3*. Among these genes, the most highly up-regulated was the amino-acid permease INDA1 (NCU07129), which was modulated by approximately 200-fold (log_2_ = 7.82 in high- and 7.16 in low-Pi). Moreover, a high-affinity potassium transporter-1 (NCU00790), three hypothetical proteins (NCU06328, NCU08490, and NCU03240) and a sugar transporter (NCU07607) were also up-regulated in both Pi conditions.

Further, we assessed the repressive effects caused by deletion of *pac-3* in both Pi conditions. Most notably, different ATPase coding-genes, including the plasma membrane proton pump H^+^-ATPase (E1-E2 ATPase-1; NCU05046), a P-type ATPase (NCU08147), and a calcium-transporting ATPase 3 (NCU07966), all of which have central roles in fungal physiology, were found to be down-regulated. Furthermore, *pac-3* deletion was seen to induce the repression of transporters for zinc/iron (NCU02879 and NCU06132), phosphorus (NCU08325 and NCU09564), and ammonium (NCU01065).

### Influence of *pac*-3 in the Oxidation-Reduction Process

We determined that the catalase-3 (NCU00355) gene, along with other essential genes, including l-amino acid oxidase (NCU01066), a flavoprotein oxidoreductase (NCU06061), and an ornithine-N5-oxygenase (NCU07117), were repressed in both Pi conditions.

Among the genes that were up-regulated in response to *pac-3* deletion were an oxidoreductase (NCU00260), a polyketide synthase-3 (NCU04865), a norsolorinic acid reductase (NCU07723), a glutathione S-transferase-1 (NCU05780), the P-450-related genes (NCU05185, NCU09185, and NCU06327) and a dyp-type peroxidase (NCU09210).

### Differentially Expressed Genes Associated With Hydrolase Activity

Our results revealed the down-regulation of a β-(1,3)-glucanosyltransferase gene (*gel1*; NCU07253), that codes for the GEL/GAS/PHR protein, in both low- and high-Pi conditions. The lipase (NCU03639) was also down-regulated in response to *pac-3* deletion. Alternatively, one metalloprotease (NCU07200) as well as members of the glycosyl hydrolase family (NCU07355, NCU08127, NCU04395, and NCU00130) were identified as up-regulated in both Pi conditions, along with.

### Gene Modulation in Response to Pi Variation

NCU06351, which codes for a phytase-1 gene, was found to be down-regulated in conditions with high-Pi concentration. Alternatively, an alternate phytase gene, NCU03255, exhibited opposing modulation and was up-regulated. However, only phytase-1 possesses a binding motif for PAC-3 in its promoter region.

Among the several proteins modulated in response to low Pi concentration and *pac-3* deletion a large portion were identified as down-regulated while only a few were up-regulated. Specifically, we identified many genes encoding for proteins related to cell wall that were down-regulated, including a chitinase-1 protein (NCU02184), a GNAT family N-acetyltransferase (NCU04039), two non-anchored cell wall proteins, -3 and -6 (NCU07817 and NCU00586), and the glycosylhydrolase family 61-5 (NCU08760). Further, the hypothetical protein NCU01311 was determined to also be repressed, which gene is associated with pathogenesis, according to GO functional annotation. The asparagine synthetase 2 (NCU04303) was also found to be down-regulated in these experimental conditions. The genes that were identified as up-regulated in response to low Pi concentration were determined to be also be associated with the deletion of *pac-*3.

In conditions containing high Pi concentrations, a large proportion of the DEGs were identified as genes that encode for transporter proteins, specifically the ABC drug exporter AtrF (NCU08056), the multidrug resistance protein MDR (NCU07546), a major facilitator superfamily transporter (NCU06847), the MFS monocarboxylate transporter (NCU05089), and the MFS multidrug transporter (NCU06860). In high Pi conditions we observed significantly more induced genes than repressed, in response to *pac-3* deletion.

### Modulated Genes Contained the Consensus Binding Motif for PAC-3

To determine which DEGs were directly or indirectly affected by the deletion of *pac-3*, we examined which DEGs contained PAC-3 binding motifs within their 1 kb promoter region. Of the total 427 identified DEGs, 231 contained potential motifs for PAC-3 binding, 85 of which (36.8%) were found within the low Pi conditions and 62 (26.84%) in high Pi conditions. Furthermore, 84 (36.36%) DEGs with the PAC-3 putative consensus-binding site (5′-GCCARG-3′) had their expression modulated in both low and high Pi concentrations. Conversely, 196 DEGs were modulated in response to the absence of the PAC-3 even without the binding motif in their promoter region, which suggests that either indirect regulation by PAC-3 occurred or there was a PAC-3 binding motif located outside the promoter region that was analyzed (1 kb). Among these DEGs, 86 (43.88%) and 49 (25%) were determined to be modulated in low and high Pi environments, respectively; while 61 DEGs (31.12%) were identified in both culture conditions simultaneously.

### Validation of Gene Expression

**FIGURE 3:**
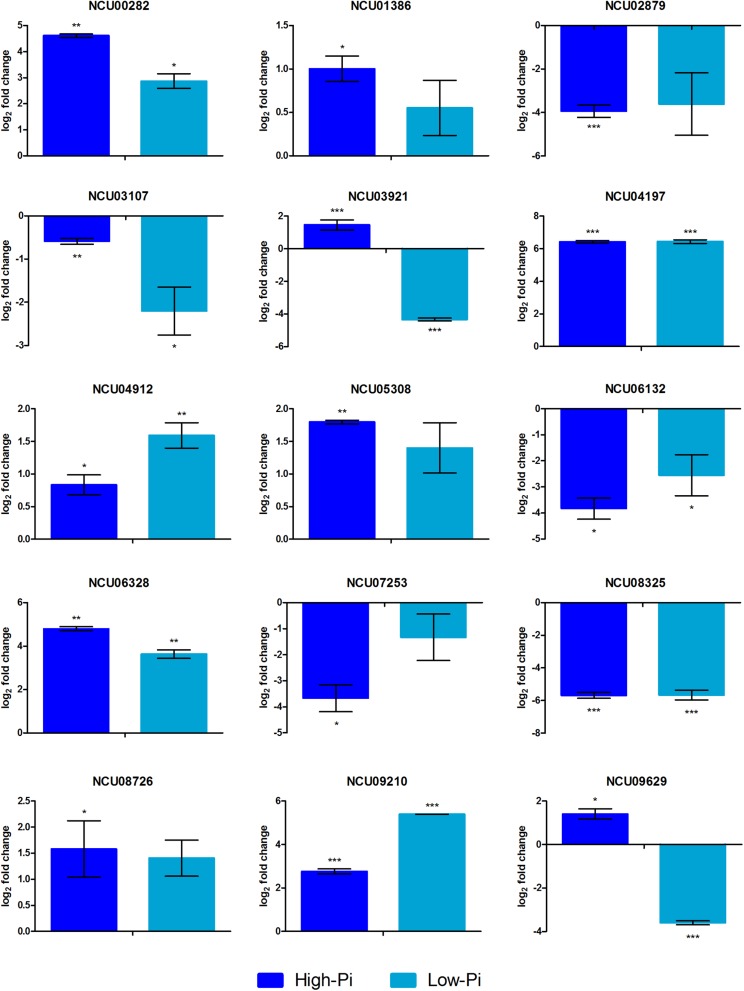
Gene expression levels represented as log_2_-fold change comparing the mutant strain Δ*pac-3* (test) to the control (Δ*mus-52* strain) in media containing low and high Pi concentrations. Asterisks indicate statistical significance determined by Student’s *t*-tests comparing treatment and control conditions at each time point (^∗^*P* < 0.05; ^∗∗^*P* < 0.01; ^∗∗∗^*P* < 0.001).

Expression analysis by qRT-PCR for the 15 selected genes using independent RNA samples was employed to validate the RNA-seq data. The results from both experiments were compared to the log_2_ ratio between the Δ*pac-3* mutant and control strains (Table 1 and Figure 3). The correlation between RNA-seq and qRT-PCR results, obtained from biological replicates, was determined to be strong and statistically significant (Pearson’s correlation, *r* = 0.953, *P* < 0.001).

**TABLE 1:** Validation of differentially expressed genes by real-time PCR (qPCR).

**ID**	**Gene product name**	**GO classification**	**Low-Pi**		**High-Pi**	
			**RNA-seq**	**qPCR**	**RNA-seq**	**qPCR**
NCU00282	Hypothetical protein	Seq-specific DNA binding RNA polymerase II transcr fact activity	3.89	2.69	4.34	4.91
NCU01386	Hypothetical protein	Zinc ion binding	–	0.53	1.50	0.97
NCU02879	Zinc/iron transporter	Zinc ion transmembrane transporter activity	–4.61	–3.52	–4.32	–3.63
NCU03107	MFS transporter		–1.79	–2.06	–	–0.51
NCU03921	Mitochondrial chaperone bcs1	ATP binding	–2.12	–4.14	2.15	1.74
NCU04197	CipC protein	Molecular function	6.62	6.64	8.44	6.52
NCU04912	HET domain-containing protein		1.94	1.50	2.21	0.94
NCU05308	Zn(II)2Cys6 transcription factor	Seq-specific DNA binding RNA polymerase II transcr fact activity	1.94	0.34	1.79	1.93
NCU06132	Siderophore iron transporter	Integral to plasma membrane	–2.98	–2.64	–2.09	–2.72
NCU06328	Hypothetical protein	Integral to membrane	4.12	3.52	4.43	4.67
NCU07253	1.3-beta-glucanosyltransferase gel1	Integral to membrane	–3.56	–0.69	–3.26	–3.33
NCU08325	Phosphorus-5	Integral to plasma membrane	–5.04	–5.75	–3.86	–5.34
NCU08726	Fluffy	Zinc ion binding	–	2.61	1.94	1.55
NCU09210	Dyp-type peroxidase	Heme binding	3.96	5.61	4.78	2.84
NCU09629	Hypothetical protein		–1.81	–3.41	2.12	1.77

*(–) not modulated at the time point.*

## Discussion

The transcription factor PAC-3 is critical component in the regulation of pH-responsive genes in fungi. As observed in various pathogenic fungi species, deletion of this transcription factor affects many cellular and molecular pathways thereby serving to interfere in the appropriate control of physiological processes essential for induction of pathogenesis (Rollins, 2003). *N. crassa* Δ*pac-3*-mutation negatively impacts on aerial hyphae growth, conidiation, and raises the production of dark pigment (Virgilio et al., 2016). These phenotypical disturbances suggest an important role for PAC-3/PacC in fungal infective success, as observed in a dermatophyte fungus (Ferreira-Nozawa et al., 2006). In *Aspergillus nidulans*, nutrient changes have been shown to modulate the transcription of the *pac-3* homolog gene, indicating the presence of interconnections between ambient pH, carbon availability, and Pi variance in a complex metabolic network (Trevisan et al., 2011; Rossi et al., 2013). Through the assessment of transcriptional changes arising from the deletion of *pac-3*, we evaluated the relevance of this transcription factor in the model fungal species *N. crassa*.

### The Import and Export of Essential Substrates Requires PAC-3

The repression of specific genes that encode proteins related to membrane transport in both high and low Pi conditions revealed a regulatory role for PAC-3 in transmembrane transport activity. Specifically, the E1-E2 ATPase-1 (ENA1), the P-type ATPase, and the calcium-transporting ATPase 3 are P-type ATPases that are involved in the active transport of cations across cellular membranes (Moller et al., 1996). As was observed in *F. oxysporum*, a functional link exists between pH signaling and the expression of the gene encoding a P-type Na^+^-ATPase. Moreover, at ambient alkaline pH, PacC serves to activate the P-Type Na^+^-ATPase *ena1* gene. This is achieved through binding of the activated form of PacC to its cognate binding sites in the promoter of *ena1*, which results in the control of ion homeostasis, a determinant for Na^+^ detoxification (Caracuel et al., 2003a).

We also determined that the ammonium transporter MEP2 was down-regulated in response to *pac-3* deletion. Since phytopathogenic fungi secrete ammonia to alkalinize the host tissue, reduced expression of MEP genes would function to directly impact fungal pathogenicity (Miyara et al., 2010).

Our results have also revealed that PAC-3 is involved in the effective transport of ions and in the homeostasis of fungal virulence. Specifically, we also observed down-regulation in the expression of a zinc/iron transporter, which is important in trafficking of metal ion substrates and in fungal homeostasis, and a siderophore-iron transporter, which functions in iron acquisition and is essential in both virulence and modulation of the plant immune system (Greenshields et al., 2007; Bailão et al., 2012; Albarouki et al., 2014). Further, expression of the H^+^-coupled *pho84/pho-5* gene, which encodes a high affinity inorganic phosphate transporter that transports manganese, zinc, cobalt, and copper ions (Jensen et al., 2003), was found to be down-regulated following the deletion of *pac-3.*

The transport-regulatory activity associated with PAC-3 seems to impact the fungal-pathogen relationship, and also symbiotic fungal associations. ATPases are thought to contribute to the uptake of Pi and other nutrients from the symbiotic interface via proton symport (Johri et al., 2015). Moreover, the acquisition of N from the soil is dependent on ammonium transporters; while the internal concentration of ions such as calcium, are responsible for the regulation of rhizobial symbiosis (Hazledine et al., 2009; Bonfante and Genre, 2010). PAC-3 serves to regulate all of these transmembrane flux processes. Furthermore, all of the genes analyzed in our study contained the PAC-3 binding motif in their promoter regions, save for the P-type ATPase (NCU08147) and the siderophore-iron transporter (NCU06132) (Supplementary Table S4).

The high-affinity transporters pho84/pho-5 and pho89/pho-4, which have been described as having Na + /Pi symporter activity (Versaw, 1995), were found to be strongly down-regulated in our study. These results suggest that PAC-3 regulates the phosphate traffic in the cell. Similarly, the *PiPT* gene, which is a *pho84* phosphate transporter homolog, from the root of the endophytic fungus *Piriformospora indica*, was reported to actively transport phosphate to the hosting plant. Further, *PiPT* knockdown mutants resulted in detrimental effects to both fungal and host, indicating that these transporters function to ameliorate the nutritional status of the host plant (Yadav et al., 2010).

Among the DEGs that were identified as being up-regulated in response to the deletion of *pac-3*, one of the strongest modulations was observed in the amino-acid permease INDA1, which has three PAC-3 binding sites within its promoter region (Supplementary Table S4). The transporter activity of INDA1, previously reported in *Trichoderma harzianum*, and expressed during development of *Rhizoctonia solani* cell walls, is thus an important factor in the mycoparasitic activity (Vasseur et al., 1995). Additionally, the expression of the high affinity potassium transporter-1, the hypothetical proteins NCU08490, with proposed Ca^2+^/Cation antiporter activity, NCU06328, identified as the integral membrane protein *pth11* (Pare et al., 2012), and NCU03240, identified as a member of the glycosyltransferase family group 2 (Wang et al., 2015), as well as a sugar transporter were all identified as being up-regulated following deletion of *pac-*3, suggesting that PAC-3 serves to coordinate the efficient acquisition of nutrients and efflux of toxic compounds.

### PAC-3 Is Involved in Secondary Metabolism, Detoxification, and Virulence

Sequencing of the *Neurospora* genome revealed several putative genes that encode proteins associated with secondary metabolite catabolism, including proteins from the polyketide synthase (PKS), non-ribosomal peptide-synthetase (NRPS) and terpenoid families (Galagan et al., 2003). Moreover, we identified DEGs whose putative products are strictly associated with fungal pathogenesis. We observed the up-regulation of the polyketide synthase-3, similar to the type I PKS from *Cochliobolus heterostrophus* (Bohnert et al., 2004), of a norsolorinic acid reductase, as well as the induction of an oxidoreductase gene, which are associated with the biosynthesis of aflatoxins, highly toxic and carcinogenic substances produced by specific fungi, including *Aspergillus flavus* and *Aspergillus parasiticus* (Yabe and Nakajima, 2004). These results suggest a role for PAC-3 in the regulation of secondary metabolism-related genes.

We also observed an up-regulation in the expression of glutathione S-transferase-1 (GST), which is associated with tolerance to oxidative stress and detoxification of a wide range of endogenous and environmental chemicals, thereby functioning to protect fungal species from plant-derived toxic metabolites. Thus, suggesting that there is an apparent regulatory effect exhibited by PAC-3 in fungal protection. GSTs are also essential for full aggressiveness of *Alternaria brassicicola* on the host plant, evidencing a role for PAC-3 in the regulation of fungal virulence (Calmes et al., 2015).

Genes that encode for the hypothetical protein NCU09185, which has potential pisatin demethylase cytochrome P450 activity, together with genes for benzoate 4-monooxygenase cytochrome P450, and a bifunctional P-450:NADPH-P450 reductase were found to be up-regulated in the absence of PAC-3. Moreover, the fungal cytochrome P450 systems are critical components in the primary and secondary metabolic pathways, and in the detoxification of xenobiotics in plant pathogenic fungi (Podobnik et al., 2008). Thus, the observed regulation via PAC-3 in *N. crassa* suggests a role for this transcription factor in plant-pathogen interactions.

A previous study has reported extracellular peroxidase activity in *Thanatephorus cucumeris*, capable of degrading lignin through the dyp-type peroxidase activity (Sugano, 2009). In our results, the up-regulation of a similar peroxidase that has been shown to be active in cellular oxidant detoxification, and to also be associated with enzymatic functions including hydrolase activity, demonstrates a potential role for PAC-3 in regulating the decomposition of extracellular compounds.

Deletion of *pac-*3 also resulted in the down-regulation of *cat3* gene, which encodes an enzyme essential for detoxification of reactive oxygen species (ROS) such as hydrogen peroxide (H_2_O_2_). Plants produce hydrogen peroxide as a mechanism of defense during pathogen interactions; thus, the observed repression in the expression of this catalase enzyme would affect the success of the pathogenic process (Tondo et al., 2010).

Furthermore, the down-regulation in the expression of L-amino acid oxidase, a flavoprotein that catalyzes the oxidative deamination of the L-amino acid α-amino group resulting in subsequent release of H_2_O_2_ and ammonia (Nuutinen and Timonen, 2008), suggests a perturbation in the fungal infection progress, since release of ammonia and H_2_O_2_ serves to propagate fungal spread.

### Differential Hydrolase Activity Affects Virulence and Activates Compensatory Enzymes

Fungal pathogens secrete a myriad of extracellular enzymes, which are assumed to be involved propagating in host infection. Specifically, in *Fusarium graminearum*, a destructive pathogen of cereals, reduced extracellular lipolytic activity was seen to reduce virulence (Voigt et al., 2005). Herein, we revealed that repression of the hydrolytic-related encoding enzyme lipase, also suggests a role for PAC-3 in fungal virulence, thereby affecting host-fungal interactions.

The β-(1,3)-glucanosyltransferase gene (*gel1*), of the glycosyl hydrolase family GH72, was identified as significantly down-regulated in the Δ*pac-3* mutant in both low and high Pi cultures. This enzyme is related to cell wall biogenesis through the incorporation of nascent β−1,3−glucan molecules into the existing β−glucan network (Kamei et al., 2013). In *F. oxysporum*, the β-1,3-glucanosyltransferase (*gas1*) is required for virulence in tomato plants, and in *Candida albicans*, the orthologue gene (*PHR1*) was reported to be pH-regulated. These enzymes have crucial roles in cell wall assembly, thus serving to maintain hyphal growth and the maintenance of the morphological state, which is imperative for its adhesive and invasive pathogenic properties (Calderon et al., 2010). Our data, therefore, highlights the direct regulatory activity that PAC-3 has on cell wall integrity, which was supported by the presence of a PAC-3 consensus sequence in the *gel1* promoter region (Supplementary Table S3). As previously observed, Rim101/PacC is required for fungal virulence and functions to positively control cell wall functions in *C. albicans* (Nobile et al., 2008). Conversely, a different beta-1,3-glucanosyltransferase was found to be up-regulated in response to *pac-3* deletion. However, since *gel1* contains a PAC-3 motif in its promoter region, and the other modulated glucanosyltransferase does not, we hypothesize that the up-regulation of this alternate enzyme is a means by which the fungus is compensating for the loss of PAC-3 function.

Among the up-regulated genes, we also identified an extracellular metalloprotease. In *Fusarium oxysporum f.* sp. *lycopersici*, a metalloprotease acts synergistically with a serine protease to cleave host chitinases thereby preventing their activity in the degradation of fungal cell wall (Jashni et al., 2015). Moreover, the hydrolytic activity of secreted proteases serves to promote the colonization and growth of the pathogen. Therefore, the induction of the metalloprotease gene suggests a protective function, acting to enhance fungal virulence thereby compensating for *pac-3* deletion.

Genes that code for glycosyl hydrolases were also found to be up-regulated as a consequence of *pac-3* deletion. Rim101/PacC have been previously shown to differentially regulate the expression of genes involved in the synthesis of specific cell wall components, including glycosyl hydrolases (Franco-Frias et al., 2014). The hydrolase-related genes found to be up-regulated in our study, may therefore, suggest the occurrence of noticeable changes in cell wall structure to compensate for the absence of PAC-3.

### Deletion of *pac*-3 Modulates Genes According to Pi Availability

The predominant form of organic phosphorus present in soils is phytate, which must be dephosphorylated via phytase and phosphatase activity before being utilized by the plants. Plants naturally produce phytases (*myo*-inositol hexakisphosphate phosphohydrolases), however, the lack of adequate levels of extracellular phytases compromises the acquisition of phosphorous. Microorganisms, principally fungi, are capable of favoring phytate-phosphorus acquisition by plants (Wyss et al., 1999; Singh and Satyanarayana, 2011). Our results revealed down-regulation of the phytase-1 gene in environments with high-Pi, thereby confirming its phosphatase activity, which is unnecessary in the presence of free Pi. Moreover, the 3-phytase A gene was found to be up-regulated in high Pi conditions. However, only the phytase-1 protein was found to contain a PAC-3 binding motif in its promoter region and thus, the induction of a 3-phytase A gene, was not regulated by the presence of PAC-3 motif.

Further, the expression of the asparagine synthetase 2 (*asn−2*), which is a notoriously non-Pi−repressible gene, was found to be down-regulated in low Pi conditions in response to deletion of *pac-3*. This enzyme is involved in *de novo* biosynthesis of nucleotides and amino acids. Within an alternate study, similar patterns of repressiveness were observed in the Δ*mak−2* strain, irrespective of the supply of Pi (Gras et al., 2013; Mendes et al., 2016). Therefore, Pi-repressiveness is potentially affected by both *pac-3* and *mak−2* deletions in *N. crassa*.

Many of the DEGs found to be down-regulated in response to *pac-*3 deletion and low-Pi were those that encode proteins related to cell wall development, including chitinase-1, a member of the GNAT N-acetyltransferase family, the non-anchored cell wall proteins -3 and -6, and the glycosylhydrolase family 61-5. As demonstrated in various fungi, fungal development, cell wall-degradation, and virulence genes are regulated by PacC/Rim101 (Caracuel et al., 2003b; Rollins, 2003; Zhang et al., 2013; Franco-Frias et al., 2014). The Δ*pac-3* mutant revealed that PAC-3 also serves to mediate adaptations in *N. crassa* within Pi-restrictive conditions through changes in cell wall biosynthesis.

Conversely, in high Pi environments, we observed many up-regulated genes in response to both the Pi condition and the absence of PAC-3. Several up-regulated genes encoding for transporter proteins were identified as being associated with drug export, multidrug resistance, and monocarboxylate transport. *Neurospora* contains a notably high number of transporter systems, which function in the secretion of secondary metabolites, the regulation of resistance to plant-produced secondary metabolites or other toxic compounds, or in the flow of signaling molecules (Borkovich et al., 2004). Here, we identified a relationship between Pi active transport and *pac-3* deletion, thereby associating PAC-3 with Pi homeostasis.

## Conclusion

We have generated a robust dataset, which advances our knowledge on regulatory mechanisms of PAC-3 within a fungus-host system. We found that in a mutually beneficial, symbiotic fungus-plant relationship (as occurs during interactions in mycorrhizal symbiosis) or during a pathogenic interaction, PAC-3 functions to regulate ambient pH while also affecting myriad of physiological functions, including adaptation to nutritional conditions, regulation of virulence, or regulating the transcription of genes associated with structural and metabolic features. Herein, we highlight the role of PAC-3 in Pi adaptation, acting as a critical regulator of environmental challenges. We hypothesize that the widespread regulatory activity of PAC-3 in fungal physiology indicates its role in the propagation of successful infections within hosts.

## Data Availability

The RNA-seq data are available at the GEO database with accession number GSE132373.

## Author Contributions

MM performed the experimental procedures and drafted the manuscript. PS performed all computational and statistical analyses. EG supported the drafting of the manuscript. WP performed some experimental procedures. RS revised the manuscript. MB revised the manuscript and provided fungal lineages. AR and NM-R designed the project, supervised the research, and prepared the manuscript. All authors participated in data analysis and interpretation, read, critically revised the manuscript, and approved the final version.

## Conflict of Interest Statement

The authors declare that the research was conducted in the absence of any commercial or financial relationships that could be construed as a potential conflict of interest.

## Footnotes

1 ftp://ftp.broadinstitute.org/pub/annotation/fungi/neurospora_crassa/assembly/NC12/
