# Supplementary material for: The pH Signaling Transcription Factor PAC-3 Regulates Metabolic and Developmental Processes in Pathogenic Fungi
Source: Front Microbiol. 2019 Sep 4;10:2076. doi: 10.3389/fmicb.2019.02076 (PMC6738131; doi:10.3389/fmicb.2019.02076)
Supplement: Supplementary file 1 [file Table_1.DOCX]

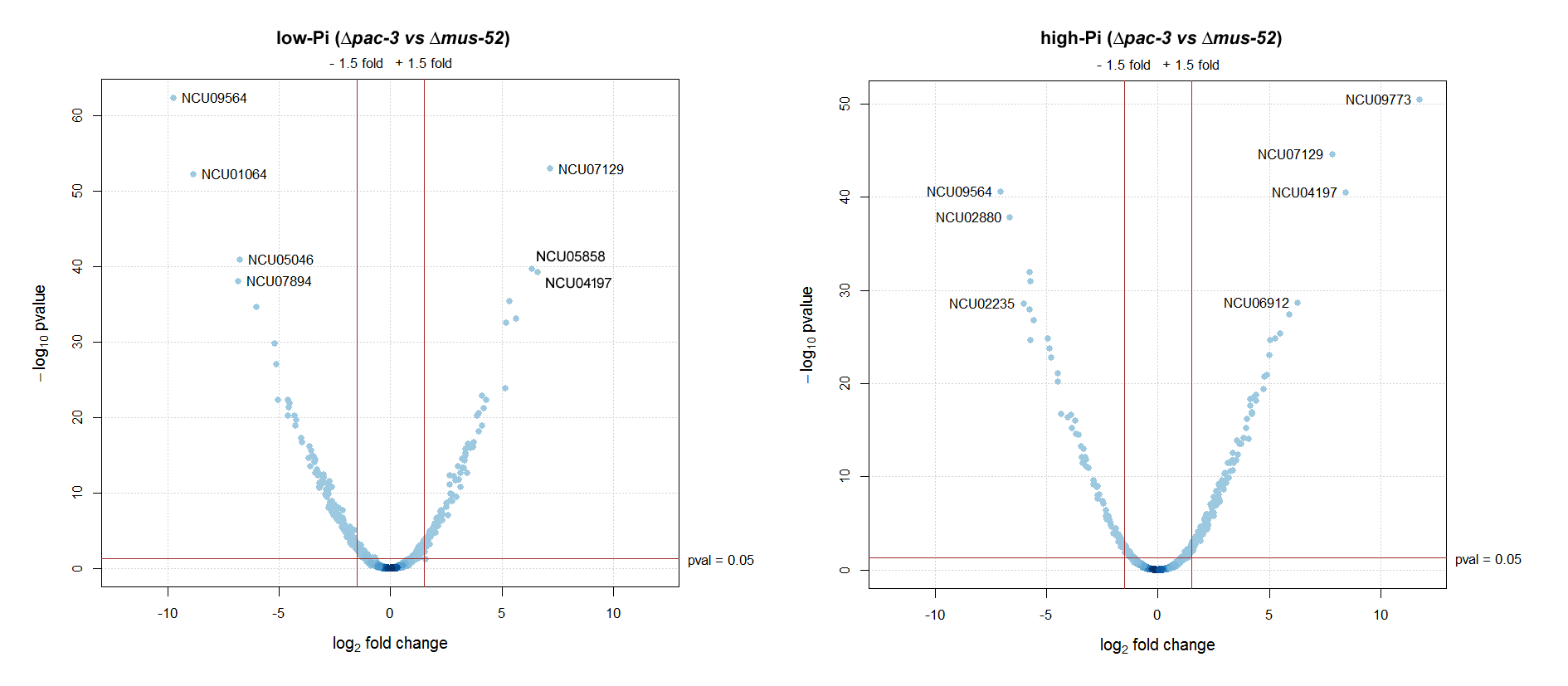


**Supplementary Figure S1**. Volcano plot depicting DEGs identified in low and high Pi conditions. The log2 fold change for DEGs are plotted against the log10 P-values under each condition. Gene names are highlighted for those with log2 fold change > 6.0.
