# Supplementary material for: The pH Signaling Transcription Factor PAC-3 Regulates Metabolic and Developmental Processes in Pathogenic Fungi
Source: Front Microbiol. 2019 Sep 4;10:2076. doi: 10.3389/fmicb.2019.02076 (PMC6738131; doi:10.3389/fmicb.2019.02076)
Supplement: Supplementary file 2 [file Table_2.DOCX]

**Supplementary Table S1.** Primers for quantitative Real-time PCR analysis.

| **ID** | **Gene Product Name** |  | **Primers (5' - 3')** | **Efficiency (%)** | **Concentration (nM)** |
| --- | --- | --- | --- | --- | --- |
| NCU06328 | hypothetical protein | FW: | GGGCATGATAGCAAGAACAAGT | 107.15 | 700 |
|  |  | REV: | AAACTCATCCGACTCATCATCC |  |  |
| NCU03921 | mitochondrial chaperone bcs1 | FW: | AAAAGACCGACATCACTGCAC | 106.37 | 700 |
|  |  | REV: | GGGAACAACTTGAACCCTGAT |  |  |
| NCU01386 | hypothetical protein | FW: | GCGGCTACATTTTCAGAAGG | 101.21 | 700 |
|  |  | REV: | GCATGGGTCGTTTGTAGTCC |  |  |
| NCU09210 | dyp-type peroxidase | FW: | GATGGCAAGCATCCTACTGAG | 101.37 | 600 |
|  |  | REV: | GATGGATGGCGTGAAGAAGTA |  |  |
| NCU00282 | hypothetical protein | FW: | GCGGCCATCTATATGGTGAA | 95.12 | 500 |
|  |  | REV: | GGGGTCGAAGGTTAGGAAGT |  |  |
| NCU05308 | Zn(II)2Cys6 transcription factor | FW: | GGAAGGATCCGCTGTTTGT | 100.62 | 500 |
|  |  | REV: | AACTCGCTCAAAACCTGCAC |  |  |
| NCU02879 | zinc/iron transporter | FW: | TTACCGTCATCAAGCCTGTCT | 97.71 | 500 |
|  |  | REV: | CTGTTTGAGTACGCCGATACC |  |  |
| NCU04197 | CipC protein | FW: | GCACTTGTGGGAGAAAGAACA | 102.20 | 600 |
|  |  | REV: | ATCCACCTTTCCCTCCAATC |  |  |
| NCU08325 | phosphorus-5 | FW: | GATGAACCACGTGTTGGAGAT | 108.56 | 600 |
|  |  | REV: | TCCTCCTCACCACTCATATCG |  |  |
| NCU06132 | siderophore iron transporter | FW: | ATCTCAATGGTGTCGGTCTTG | 98.39 | 400 |
|  |  | REV: | ACATTGTCGAGCTTGATGTCC |  |  |
| NCU04912 | HET domain-containing protein | FW: | TGCAAGGATACAGTCATGGTG | 104.60 | 500 |
|  |  | REV: | ATCCCTCTCCCTCACCAACT |  |  |
| NCU03107 | MFS transporter | FW: | GATGAGGGGAGTACGTTGGTT | 99.83 | 500 |
|  |  | REV: | TTTCTTTATCGTCCCGTACCC |  |  |
| NCU09629 | hypothetical protein | FW: | GTGGTAGGGCAGCTGGATTAT | 100.04 | 500 |
|  |  | REV: | CAACATCATTGCCAACTCTCC |  |  |
| NCU07253 | 1,3-beta-glucanosyltransferase gel1 | FW: | ACAACGCTGCTGGCTTTTC | 100.34 | 400 |
|  |  | REV: | CGACAAGGGTGAAGAGGAGA |  |  |
| NCU08726 | fluffy | FW: | CATGATGCTTCGGAGAGAGAG | 101.66 | 500 |
|  |  | REV: | CATGGATGCTAGTTGCAGTGA |  |  |
| NCU04173 | Actin | FW: | ATGTCGACGTCAGGAAGGATCT | 99.5 | 400 |
|  |  | REV: | GAGCAGTGATCTCCTTCTGCAT |  |  |
| NCU04054 | Tubulin | FW: | GCAACGGTCGTTACCTCACCT | 93.65 | 400 |
|  |  | REV: | TTCTGAACGTTGCGCATCTGG |  |  |
