# Supplementary material for: The pH Signaling Transcription Factor PAC-3 Regulates Metabolic and Developmental Processes in Pathogenic Fungi
Source: Front Microbiol. 2019 Sep 4;10:2076. doi: 10.3389/fmicb.2019.02076 (PMC6738131; doi:10.3389/fmicb.2019.02076)
Supplement: Supplementary file 3 [file Table_3.DOCX]

**Supplementary Table S2.** General features of RNA-seq reads mapped to the *N. crassa* OR74A reference genome.

| **Sample** | **Raw reads** | **High-quality reads** | **Mapped reads Bowtie2** | **Total mapped reads (%)** |
| --- | --- | --- | --- | --- |
| Δ*mus-52*low-Pi I | 31,554,520 | 27,266,844 | 23,397,679 | 85.81 |
| Δ*mus-52* low-Pi II | 20,822,501 | 18,066,751 | 15,477,786 | 85.67 |
| Δ*mus-52* low-Pi III | 14,358,702 | 12,559,482 | 10,758,452 | 85.66 |
| Δ*mus-52* high-Pi I | 46,678,086 | 40,787,870 | 34,486,144 | 84.55 |
| Δ*mus-52* high-Pi II | 20,028,090 | 17,447,064 | 14,706,130 | 84.29 |
| Δ*mus-52* high-Pi III | 29,446,490 | 25,751,431 | 21,873,265 | 84.94 |
| Δ*pac-3* low-Pi I | 57,893,077 | 50,154,831 | 42,636,622 | 85.01 |
| Δ*pac-3* low-Pi II | 14,101,111 | 12,216,765 | 10,488,093 | 85.85 |
| Δ*pac-3* low-Pi III | 18,194,364 | 15,978,729 | 13,669,803 | 85.55 |
| Δ*pac-3* high-Pi I | 19,512,177 | 17,270,545 | 14,750,772 | 85.41 |
| Δ*pac-3* high-Pi II | 19,855,856 | 17,715,842 | 15,267,513 | 86.18 |
| Δ*pac-3* high-Pi III | 22,982,762 | 20,153,356 | 17,263,365 | 85.66 |
