# Supplementary material for: The pH Signaling Transcription Factor PAC-3 Regulates Metabolic and Developmental Processes in Pathogenic Fungi
Source: Front Microbiol. 2019 Sep 4;10:2076. doi: 10.3389/fmicb.2019.02076 (PMC6738131; doi:10.3389/fmicb.2019.02076)
Supplement: Supplementary file 4 [file Table_4.DOCX]

**Supplementary Table S3.** Genes in *N. crassa* that were modulated in response to the mutant Δ*pac-3* (test) strain compared to the control (Δ*mus-52* strain) in media containing low and high Pi concentrations.

| **ID** | **low-Pi** | **high-Pi** | **Gene Product Name** |
| --- | --- | --- | --- |
| NCU04991 | 5.17 |  | oligopeptide transporter (2941 nt) |
| NCU08516 | 4.11 |  | aldose 1-epimerase (1773 nt) |
| NCU00762 | 3.46 |  | glycosylhydrolase family 5-1 (2075 nt) |
| NCU09596 | 3.23 |  | phytanoyl-CoA dioxygenase (1373 nt) |
| NCU06123 | 3.15 |  | phosphoketolase (3890 nt) |
| NCU08487 | 2.96 |  | hypothetical protein (5545 nt) |
| NCU02061 | 2.94 |  | hypothetical protein (1451 nt) |
| NCU05535 | 2.71 |  | hypothetical protein (1614 nt) |
| NCU05822 | 2.59 |  | hypothetical protein (2601 nt) |
| NCU08699 | 2.32 |  | blue light-induced-4 (2106 nt) |
| NCU08164 | 2.25 |  | retinol dehydrogenase 13 (1951 nt) |
| NCU00999 | 2.24 |  | hypothetical protein (1306 nt) |
| NCU09719 | 2.22 |  | hypothetical protein (1317 nt) |
| NCU07622 | 2.07 |  | hypothetical protein (2563 nt) |
| NCU07338 | 2.05 |  | alpha-1,6-mannosyltransferase Och1 (2257 nt) |
| NCU06079 | 2.02 |  | hypothetical protein (2418 nt) |
| NCU06416 | 1.91 |  | thymine dioxygenase (1694 nt) |
| NCU00408 | 1.90 |  | hypothetical protein (1497 nt) |
| NCU09868 | 1.90 |  | hypothetical protein (873 nt) |
| NCU04036 | 1.76 |  | hypothetical protein (1230 nt) |
| NCU01347 | 1.76 |  | hypothetical protein (1801 nt) |
| NCU17116 | 1.74 |  | hypothetical protein (1248 nt) |
| NCU02722 | 1.69 |  | hypothetical protein (1526 nt) |
| NCU00904 | 1.66 |  | D-lactate dehydrogenase (2449 nt) |
| NCU02299 | 1.64 |  | NADP-dependent leukotriene B4 12-hydroxydehydrogenase (2073 nt) |
| NCU01380 | 1.64 |  | hypothetical protein (1809 nt) |
| NCU08367 | 1.63 |  | hypothetical protein (3639 nt) |
| NCU08886 | 1.63 |  | amidohydrolase (2045 nt) |
| NCU02192 | 1.59 |  | hypothetical protein (1142 nt) |
| NCU07745 | 1.57 |  | hypothetical protein (4913 nt) |
| NCU03735 | 1.54 |  | hypothetical protein (2587 nt) |
| NCU05575 | 1.52 |  | hypothetical protein (5418 nt) |
| NCU09830 | 1.52 |  | menadione-induced gene-12 (4364 nt) |
| NCU03331 | 1.51 |  | hypothetical protein (2442 nt) |
| NCU10351 | -1.50 |  | pyridoxine kinase (2681 nt) |
| NCU08986 | -1.50 |  | hypothetical protein (1576 nt) |
| NCU03647 | -1.51 |  | hypothetical protein (4264 nt) |
| NCU00868 | -1.52 |  | hypothetical protein (1785 nt) |
| NCU03741 | -1.52 |  | NAD kinase/ATP NAD kinase (3141 nt) |
| NCU11098 | -1.52 |  | UPF0052 domain-containing protein (2517 nt) |
| NCU07931 | -1.53 |  | glyoxylate reductase (2276 nt) |
| NCU00232 | -1.54 |  | hypothetical protein (3642 nt) |
| NCU02142 | -1.55 |  | hypothetical protein (2411 nt) |
| NCU02773 | -1.55 |  | hypothetical protein (1699 nt) |
| NCU09994 | -1.55 |  | hypothetical protein (2544 nt) |
| NCU02184 | -1.56 |  | chitinase-1 (5792 nt) |
| NCU09474 | -1.57 |  | acetyltransferase (1796 nt) |
| NCU09566 | -1.57 |  | hypothetical protein (5070 nt) |
| NCU06812 | -1.57 |  | DDHD domain-containing protein (4131 nt) |
| NCU01311 | -1.57 |  | hypothetical protein (2669 nt) |
| NCU01147 | -1.58 |  | tyrosine decarboxylase (2459 nt) |
| NCU08674 | -1.58 |  | pentatricopeptide repeat protein (4983 nt) |
| NCU05616 | -1.59 |  | arsenite S-adenosylmethyltransferase (1513 nt) |
| NCU01510 | -1.60 |  | meiotically up-regulated 190 protein (5483 nt) |
| NCU05376 | -1.61 |  | p450 monooxygenase (1709 nt) |
| NCU09085 | -1.61 |  | cyclin (2529 nt) |
| NCU05526 | -1.62 |  | lysine-5 (3114 nt) |
| NCU03684 | -1.62 |  | hypothetical protein (2099 nt) |
| NCU04039 | -1.62 |  | GNAT family N-acetyltransferase (1767 nt) |
| NCU08791 | -1.64 |  | catalase-1 (2931 nt) |
| NCU09817 | -1.64 |  | aromatic-7 (2090 nt) |
| NCU07156 | -1.65 |  | histidine-6 (2094 nt) |
| NCU03170 | -1.66 |  | molybdopterin-converting factor subunit 2 (1209 nt) |
| NCU00796 | -1.66 |  | hydantoinase (3819 nt) |
| NCU00003 | -1.66 |  | hypothetical protein (1742 nt) |
| NCU01412 | -1.67 |  | proline-3 (5495 nt) |
| NCU07926 | -1.67 |  | glutaminyl-tRNA synthetase (2604 nt) |
| NCU08390 | -1.68 |  | hypothetical protein (862 nt) |
| NCU09821 | -1.68 |  | oxidoreductase (2229 nt) |
| NCU04249 | -1.69 |  | hypothetical protein (1922 nt) |
| NCU00430 | -1.69 |  | Na(+)/H(+) antiporter 2 (4654 nt) |
| NCU03158 | -1.69 |  | alpha/beta hydrolase (1581 nt) |
| NCU11414 | -1.70 |  | plasma membrane zinc ion transporter (2385 nt) |
| NCU05954 | -1.70 |  | hypothetical protein (2636 nt) |
| NCU05516 | -1.71 |  | biotin apo-protein ligase (3639 nt) |
| NCU02136 | -1.71 |  | transaldolase (2818 nt) |
| NCU04736 | -1.71 |  | calcium P-type ATPase-2 (5950 nt) |
| NCU02677 | -1.72 |  | arginine-3 (5554 nt) |
| NCU04256 | -1.73 |  | hypothetical protein (2423 nt) |
| NCU04303 | -1.73 |  | asparagine synthetase 2 (3301 nt) |
| NCU04946 | -1.73 |  | hypothetical protein (1296 nt) |
| NCU04641 | -1.73 |  | FAD dependent oxidoreductase (2072 nt) |
| NCU08621 | -1.73 |  | hypothetical protein (1606 nt) |
| NCU04246 | -1.74 |  | hypothetical protein (1779 nt) |
| NCU00522 | -1.76 |  | cystathionine beta-lyase (1859 nt) |
| NCU06290 | -1.76 |  | hypothetical protein (2925 nt) |
| NCU05377 | -1.77 |  | integral membrane protein (3359 nt) |
| NCU00802 | -1.77 |  | hypothetical protein (2026 nt) |
| NCU03367 | -1.78 |  | hypothetical protein (2651 nt) |
| NCU03965 | -1.78 |  | catabolite repression protein creC (3439 nt) |
| NCU04777 | -1.79 |  | PrnX protein (2542 nt) |
| NCU03107 | -1.79 |  | MFS transporter (2638 nt) |
| NCU00090 | -1.80 |  | pH-response transcription factor pacC/RIM101 (3194 nt) |
| NCU02397 | -1.80 |  | isoleucine-valine-5 (2543 nt) |
| NCU04415 | -1.82 |  | hypothetical protein (2874 nt) |
| NCU01454 | -1.82 |  | mitochondrial hydrolase (1907 nt) |
| NCU05617 | -1.83 |  | hypothetical protein (2322 nt) |
| NCU02124 | -1.84 |  | dienelactone hydrolase (1687 nt) |
| NCU11129 | -1.86 |  | carboxypeptidase s (2323 nt) |
| NCU04942 | -1.89 |  | methionine permease (2235 nt) |
| NCU06759 | -1.89 |  | hypothetical protein (3417 nt) |
| NCU00397 | -1.90 |  | hypothetical protein (1283 nt) |
| NCU09874 | -1.91 |  | hypothetical protein (3200 nt) |
| NCU10144 | -1.92 |  | SAM-dependent methyltransferase (1891 nt) |
| NCU02203 | -1.92 |  | C2H2 finger domain-containing protein (3311 nt) |
| NCU08880 | -1.92 |  | neutral amino acid permease (2847 nt) |
| NCU07332 | -1.92 |  | hypothetical protein (2032 nt) |
| NCU06360 | -1.93 |  | histidinol-phosphate aminotransferase (1685 nt) |
| NCU04016 | -1.93 |  | phosphoglycerate mutase (1459 nt) |
| NCU04518 | -1.95 |  | hypothetical protein (1957 nt) |
| NCU05387 | -1.96 |  | hydrolase (2356 nt) |
| NCU09365 | -1.96 |  | hypothetical protein (2792 nt) |
| NCU00300 | -1.97 |  | G-protein-coupled receptor-5 (2562 nt) |
| NCU07791 | -1.99 |  | hypothetical protein (2391 nt) |
| NCU08425 | -2.01 |  | major facilitator superfamily transporter MFS_1 (1916 nt) |
| NCU04638 | -2.03 |  | hypothetical protein (3543 nt) |
| NCU06353 | -2.03 |  | hypothetical protein (1090 nt) |
| NCU02727 | -2.04 |  | glycine cleavage system T protein (1859 nt) |
| NCU08267 | -2.05 |  | activating signal cointegrator 1 complex subunit 3 (6720 nt) |
| NCU07925 | -2.06 |  | LRP16 (1902 nt) |
| NCU01266 | -2.07 |  | phosphoinositide-specific phospholipase C (4701 nt) |
| NCU06711 | -2.08 |  | hypothetical protein (1905 nt) |
| NCU05490 | -2.08 |  | hypothetical protein (1055 nt) |
| NCU00878 | -2.09 |  | hypothetical protein (1115 nt) |
| NCU08899 | -2.09 |  | hypothetical protein (3562 nt) |
| NCU07967 | -2.12 |  | hypothetical protein (1913 nt) |
| NCU07149 | -2.13 |  | hypothetical protein (1837 nt) |
| NCU02772 | -2.14 |  | hypothetical protein (1689 nt) |
| NCU04169 | -2.15 |  | hypothetical protein (2109 nt) |
| NCU05735 | -2.18 |  | membrane transporter (2640 nt) |
| NCU02540 | -2.20 |  | meiotic expression up-regulated protein 14 (2103 nt) |
| NCU01555 | -2.22 |  | hypothetical protein (1229 nt) |
| NCU08307 | -2.22 |  | hypothetical protein (4355 nt) |
| NCU08069 | -2.33 |  | hypothetical protein (2072 nt) |
| NCU04667 | -2.34 |  | hypothetical protein (2738 nt) |
| NCU03753 | -2.35 |  | clock-controlled gene-1 (772 nt) |
| NCU11365 | -2.35 |  | aminotransferase (2803 nt) |
| NCU11356 | -2.36 |  | phenazine biosynthesis PhzC/PhzF protein (1377 nt) |
| NCU07110 | -2.36 |  | hypothetical protein (2679 nt) |
| NCU07817 | -2.40 |  | non-anchored cell wall protein-3 (1898 nt) |
| NCU05555 | -2.42 |  | hypothetical protein (3666 nt) |
| NCU02663 | -2.42 |  | L-lysine 2,3-aminomutase (2639 nt) |
| NCU05525 | -2.44 |  | DUF833 domain-containing protein (1270 nt) |
| NCU00322 | -2.47 |  | hypothetical protein (1122 nt) |
| NCU00586 | -2.48 |  | non-anchored cell wall protein-6 (2387 nt) |
| NCU04635 | -2.53 |  | hypothetical protein (1322 nt) |
| NCU00724 | -2.54 |  | hypothetical protein (2322 nt) |
| NCU00281 | -2.57 |  | UDP-glucose,sterol transferase (4682 nt) |
| NCU04805 | -2.58 |  | hypothetical protein (2185 nt) |
| NCU04605 | -2.61 |  | hypothetical protein (2172 nt) |
| NCU05899 | -2.61 |  | flotillin domain-containing protein (2780 nt) |
| NCU07082 | -2.62 |  | aspartyl-tRNA synthetase (3482 nt) |
| NCU04924 | -2.71 |  | hypothetical protein similar to phosphatidyl synthase (3016 nt) |
| NCU04266 | -2.73 |  | hypothetical protein (892 nt) |
| NCU02596 | -2.74 |  | hypothetical protein (1216 nt) |
| NCU06376 | -2.76 |  | hypothetical protein (2396 nt) |
| NCU04314 | -2.82 |  | hypothetical protein (1708 nt) |
| NCU02939 | -2.87 |  | hypothetical protein (1170 nt) |
| NCU16370 | -2.92 |  | hypothetical protein (2820 nt) |
| NCU04260 | -2.98 |  | oxidoreductase domain-containing protein (1560 nt) |
| NCU09057 | -3.00 |  | hypothetical protein (1663 nt) |
| NCU08727 | -3.08 |  | hypothetical protein (2023 nt) |
| NCU08949 | -3.14 |  | hypothetical protein (1695 nt) |
| NCU04923 | -3.17 |  | glycerol dehydrogenase-1 (1832 nt) |
| NCU06136 | -3.19 |  | hypothetical protein (1624 nt) |
| NCU07752 | -3.25 |  | hypothetical protein (1120 nt) |
| NCU07037 | -3.44 |  | hypothetical protein (2856 nt) |
| NCU02765 | -3.51 |  | RNA binding protein (1484 nt) |
| NCU04897 | -3.67 |  | hypothetical protein (2092 nt) |
| NCU00766 | -3.97 |  | hypothetical protein (1018 nt) |
| NCU08760 | -4.21 |  | glycosylhydrolase family 61-5 (1593 nt) |

Gene expression values are expressed in log_2_ fold change between each of the conditions.

| **ID** | **low-Pi** | **high-Pi** | **Gene Product Name** |
| --- | --- | --- | --- |
| NCU09773 |  | 11.74 | oligopeptide transporter (2568 nt) |
| NCU06912 |  | 6.27 | hypothetical protein (1656 nt) |
| NCU04528 |  | 4.15 | laccase precursor (2472 nt) |
| NCU09613 |  | 3.39 | hypothetical protein (1836 nt) |
| NCU08397 |  | 3.19 | hypothetical protein (3386 nt) |
| NCU04526 |  | 3.16 | hypothetical protein (2142 nt) |
| NCU09627 |  | 3.14 | hypothetical protein (1823 nt) |
| NCU04058 |  | 3.00 | hypothetical protein (1823 nt) |
| NCU07029 |  | 2.97 | hypothetical protein (4245 nt) |
| NCU04537 |  | 2.87 | monosaccharide transporter (1753 nt) |
| NCU01754 |  | 2.79 | alcohol dehydrogenase-1 (2398 nt) |
| NCU09183 |  | 2.77 | kynureninase (1518 nt) |
| NCU02138 |  | 2.76 | hypothetical protein (2961 nt) |
| NCU03660 |  | 2.75 | FK506 suppressor Sfk1 (2073 nt) |
| NCU11338 |  | 2.72 | hypothetical protein (1588 nt) |
| NCU04342 |  | 2.69 | hypothetical protein (2151 nt) |
| NCU07405 |  | 2.67 | hypothetical protein (2169 nt) |
| NCU02328 |  | 2.63 | O-methyltransferase (2577 nt) |
| NCU05277 |  | 2.63 | hypothetical protein (547 nt) |
| NCU09832 |  | 2.61 | hypothetical protein (2820 nt) |
| NCU07030 |  | 2.59 | hypothetical protein (2076 nt) |
| NCU05160 |  | 2.59 | ATP-dependent Zn protease (1689 nt) |
| NCU04502 |  | 2.52 | hypothetical protein (1265 nt) |
| NCU00754 |  | 2.50 | multidrug resistant protein (2809 nt) |
| NCU08056 |  | 2.49 | ABC drug exporter AtrF (5387 nt) |
| NCU05001 |  | 2.48 | cycloheximide-inducible-1 (1924 nt) |
| NCU00249 |  | 2.41 | hypothetical protein (2630 nt) |
| NCU08641 |  | 2.37 | hypothetical protein (3044 nt) |
| NCU00246 |  | 2.32 | hypothetical protein (3026 nt) |
| NCU02175 |  | 2.31 | phosphatidyl inositol-specific phospholipase C (4023 nt) |
| NCU09692 |  | 2.28 | phosphatidic acid phosphatase beta (2314 nt) |
| NCU03255 |  | 2.23 | 3-phytase A (2743 nt) |
| NCU04996 |  | 2.22 | hypothetical protein (1167 nt) |
| NCU09914 |  | 2.22 | hypothetical protein (857 nt) |
| NCU04872 |  | 2.21 | hypothetical protein (2305 nt) |
| NCU08603 |  | 2.21 | ankyrin repeat protein (6477 nt) |
| NCU01081 |  | 2.20 | hypothetical protein (2645 nt) |
| NCU07546 |  | 2.19 | multidrug resistance protein MDR (4825 nt) |
| NCU08852 |  | 2.18 | poly(ADP-ribose) polymerase (2843 nt) |
| NCU07513 |  | 2.17 | hypothetical protein (4567 nt) |
| NCU01504 |  | 2.15 | calcineurin binding protein (1860 nt) |
| NCU02086 |  | 2.13 | hypothetical protein (3044 nt) |
| NCU08281 |  | 2.12 | hypothetical protein (3754 nt) |
| NCU05141 |  | 2.07 | hypothetical protein (1671 nt) |
| NCU07241 |  | 2.05 | hypothetical protein (3283 nt) |
| NCU05897 |  | 2.03 | l-fucose permease (2525 nt) |
| NCU09771 |  | 2.02 | DUF895 domain membrane protein (2293 nt) |
| NCU09335 |  | 2.02 | hypothetical protein (3840 nt) |
| NCU10246 |  | 1.97 | hypothetical protein (1095 nt) |
| NCU08820 |  | 1.96 | hypothetical protein (5083 nt) |
| NCU06847 |  | 1.95 | major facilitator superfamily transporter (3117 nt) |
| NCU09209 |  | 1.95 | galactose oxidase (2544 nt) |
| NCU06875 |  | 1.95 | hypothetical protein (2858 nt) |
| NCU08726 |  | 1.94 | fluffy (3489 nt) |
| NCU08229 |  | 1.92 | hypothetical protein (2251 nt) |
| NCU02628 |  | 1.91 | hypothetical protein (4606 nt) |
| NCU07017 |  | 1.90 | hypothetical protein (1695 nt) |
| NCU06337 |  | 1.88 | hypothetical protein (2425 nt) |
| NCU07491 |  | 1.87 | hypothetical protein (2125 nt) |
| NCU00604 |  | 1.86 | hypothetical protein (2396 nt) |
| NCU04493 |  | 1.85 | hypothetical protein (3118 nt) |
| NCU07257 |  | 1.82 | F-box domain-containing protein (3057 nt) |
| NCU05089 |  | 1.82 | MFS monocarboxylate transporter (2065 nt) |
| NCU09693 |  | 1.75 | hypothetical protein (1790 nt) |
| NCU04543 |  | 1.71 | hypothetical protein (1600 nt) |
| NCU09069 |  | 1.70 | hypothetical protein (2487 nt) |
| NCU05315 |  | 1.69 | hypothetical protein (3304 nt) |
| NCU01423 |  | 1.69 | hypothetical protein (1839 nt) |
| NCU00240 |  | 1.68 | hypothetical protein (3482 nt) |
| NCU09782 |  | 1.67 | hypothetical protein (2896 nt) |
| NCU06860 |  | 1.67 | MFS multidrug transporter (2508 nt) |
| NCU02989 |  | 1.67 | hypothetical protein (2089 nt) |
| NCU09504 |  | 1.67 | hypothetical protein (2610 nt) |
| NCU03355 |  | 1.66 | calpain-5 (3556 nt) |
| NCU02191 |  | 1.63 | hypothetical protein (2572 nt) |
| NCU04554 |  | 1.63 | glycosylhydrolase family 18-5 (2107 nt) |
| NCU09020 |  | 1.62 | hypothetical protein (2914 nt) |
| NCU16477 |  | 1.59 | hypothetical protein (1261 nt) |
| NCU08847 |  | 1.57 | hypothetical protein (3924 nt) |
| NCU05079 |  | 1.56 | MFS peptide transporter (2703 nt) |
| NCU03650 |  | 1.55 | DNA repair protein RAD16 (4554 nt) |
| NCU00262 |  | 1.53 | hypothetical protein (1873 nt) |
| NCU08155 |  | 1.53 | hypothetical protein (1395 nt) |
| NCU08816 |  | 1.52 | hypothetical protein (2234 nt) |
| NCU02482 |  | 1.52 | tricarboxylic acid-2 (3070 nt) |
| NCU10721 |  | 1.52 | solute carrier family 35 member B1 protein (1902 nt) |
| NCU05828 |  | 1.52 | hypothetical protein (3759 nt) |
| NCU01340 |  | 1.52 | hypothetical protein (1091 nt) |
| NCU01386 |  | 1.50 | hypothetical protein (3265 nt) |
| NCU05770 |  | 1.50 | catalase-2 (2969 nt) |
| NCU02990 |  | 1.50 | hypothetical protein (2240 nt) |
| NCU09308 |  | -1.50 | glycoprotease (2270 nt) |
| NCU08225 |  | -1.51 | high affinity nickel transporter nic1 (3420 nt) |
| NCU06666 |  | -1.55 | inositol (2856 nt) |
| NCU02167 |  | -1.57 | Krev-1-like (2783 nt) |
| NCU02174 |  | -1.60 | hypothetical protein (1980 nt) |
| NCU06351 |  | -1.62 | phytase-1 (2832 nt) |
| NCU04132 |  | -1.64 | hypothetical protein (2561 nt) |
| NCU17088 |  | -1.65 | aspartyl aminopeptidase (2273 nt) |
| NCU08005 |  | -1.65 | NADPH-adrenodoxin reductase Arh1 (2245 nt) |
| NCU08045 |  | -1.70 | choline-1 (3848 nt) |
| NCU02214 |  | -1.73 | tall aerial hyphae-2 (3199 nt) |
| NCU00399 |  | -1.79 | cell wall protein PhiA (1971 nt) |
| NCU07046 |  | -1.79 | hypothetical protein (1626 nt) |
| NCU03802 |  | -1.83 | carnitine biosynthesis-1 (2551 nt) |
| NCU00676 |  | -1.91 | F1-ATP synthase assembly protein (1727 nt) |
| NCU01931 |  | -1.98 | hypothetical protein (2334 nt) |
| NCU09909 |  | -2.18 | urea active transporter (2823 nt) |
| NCU16673 |  | -2.90 | hypothetical protein (1435 nt) |
| NCU00077 |  | -5.73 | mutagen sensitive-52 (3425 nt) |
| NCU02235 |  | -6.03 | glycosylhydrolase family 47-6 (4014 nt) |

Gene expression values are expressed in log_2_ fold change between each of the conditions.

| **ID** | **low-Pi** | **high-Pi** | **Gene Product Name** |
| --- | --- | --- | --- |
| NCU07129 | 7.16 | 7.82 | amino-acid permease inda1 (3625 nt) |
| NCU04197 | 6.62 | 8.44 | CipC protein (794 nt) |
| NCU05858 | 6.36 | 5.91 | fatty acid oxygenase (4097 nt) |
| NCU08457 | 5.64 | 4.00 | easily wettable (1894 nt) |
| NCU08739 | 5.34 | 5.27 | endothiapepsin (1988 nt) |
| NCU00790 | 5.20 | 4.99 | high affinity potassium transporter-1 (3449 nt) |
| NCU05755 | 4.31 | 4.76 | hypothetical protein (945 nt) |
| NCU07083 | 4.19 | 3.37 | hypothetical protein (2565 nt) |
| NCU06328 | 4.12 | 4.43 | hypothetical protein (2961 nt) |
| NCU10387 | 3.97 | 4.10 | dimethylaniline monooxygenase (2794 nt) |
| NCU09210 | 3.96 | 4.78 | dyp-type peroxidase (2550 nt) |
| NCU00282 | 3.89 | 4.34 | hypothetical protein (5257 nt) |
| NCU03422 | 3.73 | 3.25 | hypothetical protein (2808 nt) |
| NCU08173 | 3.71 | 1.94 | early conidial development-2 (1713 nt) |
| NCU09305 | 3.70 | 3.06 | hypothetical protein (1370 nt) |
| NCU10245 | 3.60 | 4.91 | hypothetical protein (2091 nt) |
| NCU02910 | 3.48 | 4.32 | hypothetical protein (1387 nt) |
| NCU08087 | 3.38 | 2.77 | hypothetical protein (1584 nt) |
| NCU07355 | 3.37 | 2.76 | glycosyl hydrolase family 71 protein (2337 nt) |
| NCU04865 | 3.37 | 3.42 | polyketide synthase-3 (8785 nt) |
| NCU05780 | 3.32 | 3.84 | glutathione S-transferase-1 (1123 nt) |
| NCU05908 | 3.29 | 2.75 | hypothetical protein (2167 nt) |
| NCU05105 | 3.24 | 4.41 | glucan endo-1,3-beta-glucosidase (2628 nt) |
| NCU02877 | 3.14 | 3.95 | hypothetical protein (3056 nt) |
| NCU07723 | 3.08 | 2.26 | norsolorinic acid reductase (3303 nt) |
| NCU07607 | 3.02 | 4.22 | sugar transporter (2952 nt) |
| NCU09505 | 2.84 | 3.67 | hypothetical protein (2164 nt) |
| NCU01579 | 2.78 | 2.20 | hypothetical protein (991 nt) |
| NCU09724 | 2.76 | 3.74 | hypothetical protein (1812 nt) |
| NCU02875 | 2.70 | 2.68 | hypothetical protein (3407 nt) |
| NCU03240 | 2.67 | 5.05 | hypothetical protein (3323 nt) |
| NCU06327 | 2.66 | 2.75 | benzoate 4-monooxygenase cytochrome P450 (2930 nt) |
| NCU05068 | 2.57 | 3.05 | hypothetical protein (1660 nt) |
| NCU08418 | 2.53 | 2.64 | tripeptidyl-peptidase (2150 nt) |
| NCU02363 | 2.51 | 3.60 | RTA1 domain-containing protein (1252 nt) |
| NCU04909 | 2.34 | 2.27 | hypothetical protein (1775 nt) |
| NCU06334 | 2.32 | 2.15 | hypothetical protein (3501 nt) |
| NCU00306 | 2.28 | 4.24 | MFS multidrug transporter (2796 nt) |
| NCU04046 | 2.27 | 2.98 | hypothetical protein (1887 nt) |
| NCU00260 | 2.25 | 2.15 | oxidoreductase (1834 nt) |
| NCU05832 | 2.23 | 2.19 | hypothetical protein (3095 nt) |
| NCU05185 | 2.20 | 2.60 | bifunctional P-450:NADPH-P450 reductase (3843 nt) |
| NCU02213 | 2.18 | 2.94 | hypothetical protein (2568 nt) |
| NCU04395 | 2.17 | 3.51 | glycosyl hydrolase family 30-1 (1717 nt) |
| NCU08230 | 2.15 | 1.89 | hypothetical protein (4748 nt) |
| NCU09772 | 2.13 | 3.33 | hypothetical protein (2738 nt) |
| NCU10291 | 2.13 | 1.56 | hypothetical protein (2572 nt) |
| NCU06305 | 2.13 | 2.32 | tartrate transporter (2061 nt) |
| NCU07200 | 2.09 | 2.22 | metalloprotease 1 (1672 nt) |
| NCU08738 | 2.08 | 3.24 | MFS peptide transporter (2988 nt) |
| NCU11292 | 2.07 | 2.95 | hypothetical protein (1109 nt) |
| NCU09355 | 2.02 | 2.45 | hypothetical protein (4162 nt) |
| NCU00130 | 2.00 | 2.30 | glycosylhydrolase family 1-1 (2415 nt) |
| NCU04866 | 2.00 | 2.15 | all development altered-6 (2565 nt) |
| NCU09422 | 1.99 | 1.87 | hypothetical protein (1666 nt) |
| NCU04912 | 1.94 | 2.21 | HET domain-containing protein (1824 nt) |
| NCU05308 | 1.94 | 1.79 | Zn(II)2Cys6 transcription factor (3948 nt) |
| NCU01881 | 1.87 | 2.03 | hypothetical protein (4070 nt) |
| NCU08169 | 1.84 | 1.81 | hypothetical protein (1985 nt) |
| NCU07876 | 1.82 | 1.70 | PH domain-containing protein (2746 nt) |
| NCU07923 | 1.79 | 1.60 | hypothetical protein (2676 nt) |
| NCU01769 | 1.79 | 2.44 | hypothetical protein (2406 nt) |
| NCU04963 | 1.79 | 3.55 | high-affinity glucose transporter (2367 nt) |
| NCU05395 | 1.75 | 1.66 | hypothetical protein (1088 nt) |
| NCU09185 | 1.74 | 2.44 | hypothetical protein (2297 nt) |
| NCU08117 | 1.74 | 2.13 | hypothetical protein (2557 nt) |
| NCU04442 | 1.73 | 1.77 | GAL10 (2221 nt) |
| NCU09182 | 1.73 | 2.51 | stress responsive A/B barrel domain-containing protein (868 nt) |
| NCU00161 | 1.71 | 1.86 | hypothetical protein (3841 nt) |
| NCU00304 | 1.71 | 2.18 | hypothetical protein (1951 nt) |
| NCU09559 | 1.70 | 2.12 | clock-controlled gene-9 (4672 nt) |
| NCU06239 | 1.64 | 1.57 | hypothetical protein (3413 nt) |
| NCU08490 | 1.62 | 1.97 | hypothetical protein (1690 nt) |
| NCU08055 | 1.60 | 2.45 | b-ZIP transcription factor IDI4 (1638 nt) |
| NCU01509 | 1.59 | 1.87 | hypothetical protein (3568 nt) |
| NCU01298 | 1.59 | 4.15 | hypothetical protein (1598 nt) |
| NCU07740 | 1.58 | 3.37 | hypothetical protein (2233 nt) |
| NCU04276 | 1.58 | 5.48 | hypothetical protein (1432 nt) |
| NCU08127 | 1.57 | 2.13 | glycosylhydrolase family 76-3 (2826 nt) |
| NCU08171 | 1.57 | 2.11 | anchored cell wall protein-12 (1887 nt) |
| NCU08909 | 1.56 | 2.14 | beta-1,3-glucanosyltransferase (2536 nt) |
| NCU06111 | 1.55 | 1.52 | GTPase Ras2p (2264 nt) |
| NCU08231 | 1.55 | 1.70 | acyltransferase (2057 nt) |
| NCU16992 | 1.55 | 1.57 | mating factor a-1 (1161 nt) |
| NCU04122 | 1.51 | 1.87 | malate dehydrogenase (2413 nt) |
| NCU07178 | 1.51 | 1.71 | hypothetical protein (2956 nt) |
| NCU09647 | -1.50 | -1.53 | hypothetical protein (2198 nt) |
| NCU06772 | -1.51 | -1.90 | hypothetical protein (2905 nt) |
| NCU03131 | -1.54 | -2.32 | FAD dependent oxidoreductase superfamily (1891 nt) |
| NCU04569 | -1.59 | -1.78 | 5-oxoprolinase (5282 nt) |
| NCU04936 | -1.59 | -1.63 | UDP-glucose 6-dehydrogenase (3320 nt) |
| NCU08351 | -1.65 | -2.30 | hypothetical protein (801 nt) |
| NCU09881 | -1.67 | -2.21 | hypothetical protein (2617 nt) |
| NCU08895 | -1.79 | -1.87 | PNS1 (2616 nt) |
| NCU01044 | -1.80 | -1.84 | spray (3689 nt) |
| NCU09629 | -1.81 | 2.12 | hypothetical protein (4219 nt) |
| NCU04292 | -1.85 | -1.62 | branched-chain-amino-acid aminotransferase (2433 nt) |
| NCU04433 | -1.97 | -2.74 | cysteine-14 (4414 nt) |
| NCU01781 | -2.05 | -2.16 | hypothetical protein (2575 nt) |
| NCU06957 | -2.06 | -2.07 | hypothetical protein (3095 nt) |
| NCU01897 | -2.07 | -2.45 | hypothetical protein (2049 nt) |
| NCU00155 | -2.10 | -1.86 | C6 transcription factor (5224 nt) |
| NCU03921 | -2.12 | 2.15 | mitochondrial chaperone bcs1 (3447 nt) |
| NCU06125 | -2.14 | -1.53 | hypothetical protein (2403 nt) |
| NCU09116 | -2.18 | -1.60 | aromatic aminotransferase Aro8 (4361 nt) |
| NCU05805 | -2.43 | -1.57 | serine hydroxymethyl transferase (2855 nt) |
| NCU00355 | -2.51 | -2.71 | catalase-3 (3206 nt) |
| NCU03639 | -2.54 | -3.46 | lipase (1637 nt) |
| NCU07748 | -2.62 | -4.94 | hypothetical protein (1281 nt) |
| NCU06110 | -2.64 | -3.34 | thiazole biosynthetic enzyme (2678 nt) |
| NCU01065 | -2.65 | -3.10 | ammonium transporter MEP2 (2491 nt) |
| NCU09935 | -2.72 | -2.86 | hypothetical protein (2208 nt) |
| NCU04230 | -2.74 | -1.89 | acetate utilization-3 (2923 nt) |
| NCU07953 | -2.78 | -2.47 | alternative oxidase-1 (2037 nt) |
| NCU17064 | -2.78 | -1.62 | 5-nitroimidazole antibiotic resistance protein (1725 nt) |
| NCU06061 | -2.82 | -2.34 | oxidoreductase (1995 nt) |
| NCU05518 | -2.83 | -2.88 | peroxisomal copper amine oxidase (2849 nt) |
| NCU06132 | -2.98 | -2.09 | siderophore iron transporter (2605 nt) |
| NCU07117 | -3.02 | -2.28 | ornithine-N5-oxygenase (2773 nt) |
| NCU08439 | -3.11 | -2.28 | leptomycin B resistance protein pmd1 (4777 nt) |
| NCU10038 | -3.17 | -4.04 | glycerophosphoryl diester phosphodiesterase (1645 nt) |
| NCU09345 | -3.25 | -3.70 | no message in thiamine-1 (2959 nt) |
| NCU04466 | -3.30 | -2.63 | cyanamide hydratase (1966 nt) |
| NCU02361 | -3.35 | -3.55 | formamidase (2165 nt) |
| NCU08441 | -3.38 | -2.70 | non-ribosomal peptide synthetase (6449 nt) |
| NCU07016 | -3.41 | -3.21 | hypothetical protein (2283 nt) |
| NCU03153 | -3.42 | -3.40 | hypothetical protein (2081 nt) |
| NCU07253 | -3.56 | -3.26 | 1,3-beta-glucanosyltransferase gel1 (2321 nt) |
| NCU01066 | -3.60 | -4.48 | l-amino acid oxidase (2781 nt) |
| NCU06062 | -3.63 | -2.69 | aerobactin siderophore biosynthesis protein iucB (1944 nt) |
| NCU07966 | -4.00 | -3.88 | calcium-transporting ATPase 3 (3884 nt) |
| NCU06063 | -4.26 | -3.38 | long-chain-fatty-acid-CoA ligase (2561 nt) |
| NCU08184 | -4.32 | -3.21 | hypothetical protein (836 nt) |
| NCU08183 | -4.51 | -3.65 | hypothetical protein (1341 nt) |
| NCU09271 | -4.57 | -4.85 | hypothetical protein (3242 nt) |
| NCU08147 | -4.61 | -4.50 | P-type ATPase (4301 nt) |
| NCU02879 | -4.61 | -4.32 | zinc/iron transporter (1792 nt) |
| NCU08325 | -5.04 | -3.86 | phosphorus-5 (2228 nt) |
| NCU07434 | -5.12 | -4.80 | short-chain dehydrogenase/reductase SDR (1663 nt) |
| NCU04452 | -5.18 | -5.77 | menadione-induced gene-3 (1620 nt) |
| NCU02880 | -6.00 | -6.64 | hypothetical protein (1219 nt) |
| NCU05046 | -6.77 | -5.72 | E1-E2 ATPase-1 (4936 nt) |
| NCU07894 | -6.85 | -5.55 | oligopeptide transporter 2 (6060 nt) |
| NCU01064 | -8.84 | -5.77 | hypothetical protein (877 nt) |
| NCU09564 | -9.74 | -7.06 | phosphorus-4 (4123 nt) |

Gene expression values are expressed in log_2_ fold change between each of the conditions.
