# Supplementary material for: The pH Signaling Transcription Factor PAC-3 Regulates Metabolic and Developmental Processes in Pathogenic Fungi
Source: Front Microbiol. 2019 Sep 4;10:2076. doi: 10.3389/fmicb.2019.02076 (PMC6738131; doi:10.3389/fmicb.2019.02076)
Supplement: Supplementary file 5 [file Table_5.DOCX]

**Supplementary Table S4.** Genes in *N. crassa* that were modulated in response to the mutant ∆*pac-3* (test) strain compared to the control (∆*mus-52* strain) in media containing low and high Pi concentrations. The number of putative consensus binding sites was determined by identifying the following DNA sequences upstream (1000-bp) from the promoter region of the genes: *pac-3*, 5’-GCCARG-3’.

**low-Pi (**∆***pac-3 vs*** ∆***mus-52*)**

| **ID** | **Gene Product Name** | **low-Pi** | **high-Pi** | **Consensus** |
| --- | --- | --- | --- | --- |
| NCU08516 | aldose 1-epimerase | 4.11 |  | 2 |
| NCU09596 | phytanoyl-CoAdioxygenase | 3.23 |  | 1 |
| NCU08487 | hypothetical protein | 2.96 |  | 1 |
| NCU05535 | hypothetical protein | 2.71 |  | 1 |
| NCU08699 | blue light-induced-4 | 2.32 |  | 1 |
| NCU08164 | retinol dehydrogenase 13 | 2.25 |  | 1 |
| NCU09719 | hypothetical protein | 2.22 |  | 2 |
| NCU07338 | alpha-1,6-mannosyltransferase Och1 | 2.05 |  | 1 |
| NCU04036 | hypothetical protein | 1.76 |  | 3 |
| NCU17116 | hypothetical protein | 1.74 |  | 1 |
| NCU02722 | hypothetical protein | 1.69 |  | 1 |
| NCU08367 | hypothetical protein | 1.63 |  | 2 |
| NCU02192 | hypothetical protein | 1.59 |  | 3 |
| NCU05575 | hypothetical protein | 1.52 |  | 2 |
| NCU08986 | hypothetical protein | -1.50 |  | 3 |
| NCU03647 | hypothetical protein | -1.51 |  | 3 |
| NCU03741 | NAD kinase/ATP NAD kinase | -1.52 |  | 2 |
| NCU11098 | UPF0052 domain-containing protein | -1.52 |  | 1 |
| NCU02142 | hypothetical protein | -1.55 |  | 3 |
| NCU02773 | hypothetical protein | -1.55 |  | 1 |
| NCU09994 | hypothetical protein | -1.55 |  | 1 |
| NCU02184 | chitinase-1 | -1.56 |  | 1 |
| NCU01311 | hypothetical protein | -1.57 |  | 1 |
| NCU06812 | DDHD domain-containing protein | -1.57 |  | 1 |
| NCU09566 | hypothetical protein | -1.57 |  | 1 |
| NCU01147 | tyrosine decarboxylase | -1.58 |  | 1 |
| NCU08674 | pentatricopeptide repeat protein | -1.58 |  | 2 |
| NCU05616 | arsenite S-adenosylmethyltransferase | -1.59 |  | 1 |
| NCU01510 | meiotically up-regulated 190 protein | -1.60 |  | 1 |
| NCU05376 | p450 monooxygenase | -1.61 |  | 1 |
| NCU05526 | lysine-5 | -1.62 |  | 1 |
| NCU08791 | catalase-1 | -1.64 |  | 2 |
| NCU00003 | hypothetical protein | -1.66 |  | 3 |
| NCU03170 | molybdopterin-converting factor subunit 2 | -1.66 |  | 1 |
| NCU01412 | proline-3 | -1.67 |  | 2 |
| NCU08390 | hypothetical protein | -1.68 |  | 1 |
| NCU09821 | oxidoreductase | -1.68 |  | 1 |
| NCU00430 | Na(+)/H(+) antiporter 2 | -1.69 |  | 1 |
| NCU03158 | alpha/beta hydrolase | -1.69 |  | 1 |
| NCU04249 | hypothetical protein | -1.69 |  | 1 |
| NCU04736 | calcium P-type ATPase-2 | -1.71 |  | 4 |
| NCU05516 | biotin apo-protein ligase | -1.71 |  | 1 |
| NCU02677 | arginine-3 | -1.72 |  | 2 |
| NCU04303 | asparagine synthetase 2 | -1.73 |  | 1 |
| NCU04946 | hypothetical protein | -1.73 |  | 1 |
| NCU04246 | hypothetical protein | -1.74 |  | 3 |
| NCU05377 | integral membrane protein | -1.77 |  | 1 |
| NCU03367 | hypothetical protein | -1.78 |  | 2 |
| NCU03965 | catabolite repression protein creC | -1.78 |  | 1 |
| NCU04777 | PrnX protein | -1.79 |  | 1 |
| NCU00090 | pH-response transcription factor pacC/RIM101 | -1.80 |  | 5 |
| NCU01454 | mitochondrial hydrolase | -1.82 |  | 1 |
| NCU04415 | hypothetical protein | -1.82 |  | 2 |
| NCU05617 | hypothetical protein | -1.83 |  | 2 |
| NCU04942 | methionine permease | -1.89 |  | 2 |
| NCU09874 | hypothetical protein | -1.91 |  | 2 |
| NCU02203 | C2H2 fingerdomain-containing protein | -1.92 |  | 1 |
| NCU08880 | neutral amino acid permease | -1.92 |  | 2 |
| NCU04016 | phosphoglycerate mutase | -1.93 |  | 2 |
| NCU09365 | hypothetical protein | -1.96 |  | 1 |
| NCU00300 | G-protein-coupled receptor-5 | -1.97 |  | 1 |
| NCU04638 | hypothetical protein | -2.03 |  | 2 |
| NCU05490 | hypothetical protein | -2.08 |  | 1 |
| NCU00878 | hypothetical protein | -2.09 |  | 1 |
| NCU07967 | hypothetical protein | -2.12 |  | 1 |
| NCU08069 | hypothetical protein | -2.33 |  | 1 |
| NCU11365 | aminotransferase | -2.35 |  | 1 |
| NCU02663 | L-lysine 2,3-amino mutase | -2.42 |  | 1 |
| NCU05555 | hypothetical protein | -2.42 |  | 3 |
| NCU00586 | non-anchored cell wall protein-6 | -2.48 |  | 2 |
| NCU00281 | UDP-glucose,sterol transferase | -2.57 |  | 1 |
| NCU04605 | hypothetical protein | -2.61 |  | 1 |
| NCU07082 | aspartyl-tRNA synthetase | -2.62 |  | 1 |
| NCU04924 | hypothetical protein similar to phosphatidyl synthase | -2.71 |  | 4 |
| NCU04266 | hypothetical protein | -2.73 |  | 1 |
| NCU06376 | hypothetical protein | -2.76 |  | 1 |
| NCU04314 | hypothetical protein | -2.82 |  | 3 |
| NCU02939 | hypothetical protein | -2.87 |  | 2 |
| NCU16370 | hypothetical protein | -2.92 |  | 1 |
| NCU04260 | oxidoreductase domain-containing protein | -2.98 |  | 2 |
| NCU04923 | glycerol dehydrogenase-1 | -3.17 |  | 3 |
| NCU07752 | hypothetical protein | -3.25 |  | 1 |
| NCU07037 | hypothetical protein | -3.44 |  | 1 |
| NCU04897 | hypothetical protein | -3.67 |  | 2 |
| NCU08760 | glycosylhydrolase family 61-5 | -4.21 |  | 1 |

Gene expression values are expressed in log_2_ fold change between each condition.

**high-Pi (**∆***pac-3 vs*** ∆***mus-52*)**

| **ID** | **Gene Product Name** | **low-Pi** | **high-Pi** | **Consensus** |
| --- | --- | --- | --- | --- |
| NCU06912 | Hypothetical protein |  | 6.27 | 1 |
| NCU09613 | Hypothetical protein |  | 3.39 | 3 |
| NCU09627 | Hypothetical protein |  | 3.14 | 1 |
| NCU04058 | Hypothetical protein |  | 3.00 | 1 |
| NCU07029 | Hypothetical protein |  | 2.97 | 2 |
| NCU01754 | alcohol dehydrogenase-1 |  | 2.79 | 1 |
| NCU09183 | Kynureninase |  | 2.77 | 5 |
| NCU02138 | Hypothetical protein |  | 2.76 | 3 |
| NCU11338 | Hypothetical protein |  | 2.72 | 3 |
| NCU07405 | Hypothetical protein |  | 2.67 | 1 |
| NCU09832 | Hypothetical protein |  | 2.61 | 1 |
| NCU05160 | ATP-dependent Zn protease |  | 2.59 | 1 |
| NCU07030 | Hypothetical protein |  | 2.59 | 2 |
| NCU04502 | Hypothetical protein |  | 2.52 | 4 |
| NCU00754 | Multidrugresistantprotein |  | 2.50 | 1 |
| NCU05001 | cycloheximide-inducible-1 |  | 2.48 | 1 |
| NCU08641 | Hypothetical protein |  | 2.37 | 3 |
| NCU00246 | Hypothetical protein |  | 2.32 | 1 |
| NCU09692 | Phosphatidic acid phosphatase beta |  | 2.28 | 2 |
| NCU04996 | Hypothetical protein |  | 2.22 | 2 |
| NCU09914 | Hypothetical protein |  | 2.22 | 2 |
| NCU04872 | Hypothetical protein |  | 2.21 | 1 |
| NCU07546 | Multidrug resistance protein MDR |  | 2.19 | 4 |
| NCU08852 | poly(ADP-ribose) polymerase |  | 2.18 | 4 |
| NCU07513 | Hypothetical protein |  | 2.17 | 1 |
| NCU01504 | Calcineurin binding protein |  | 2.15 | 1 |
| NCU02086 | hypothetical protein |  | 2.13 | 1 |
| NCU08281 | hypothetical protein |  | 2.12 | 1 |
| NCU09335 | hypothetical protein |  | 2.02 | 1 |
| NCU09771 | DUF895 domain membrane protein |  | 2.02 | 1 |
| NCU06847 | Major facilitator superfamily transporter |  | 1.95 | 1 |
| NCU08726 | fluffy |  | 1.94 | 3 |
| NCU02628 | hypothetical protein |  | 1.91 | 1 |
| NCU07017 | hypothetical protein |  | 1.90 | 1 |
| NCU07491 | hypothetical protein |  | 1.87 | 1 |
| NCU09693 | hypothetical protein |  | 1.75 | 1 |
| NCU04543 | hypothetical protein |  | 1.71 | 1 |
| NCU00240 | hypothetical protein |  | 1.68 | 1 |
| NCU02989 | hypothetical protein |  | 1.67 | 1 |
| NCU09782 | hypothetical protein |  | 1.67 | 1 |
| NCU03355 | calpain-5 |  | 1.66 | 1 |
| NCU04554 | Glycosyl hydrolasefamily 18-5 |  | 1.63 | 1 |
| NCU09020 | hypothetical protein |  | 1.62 | 2 |
| NCU16477 | hypothetical protein |  | 1.59 | 1 |
| NCU08847 | hypothetical protein |  | 1.57 | 1 |
| NCU05079 | MFS peptide transporter |  | 1.56 | 1 |
| NCU03650 | DNA repairprotein RAD16 |  | 1.55 | 2 |
| NCU00262 | hypothetical protein |  | 1.53 | 1 |
| NCU02990 | hypothetical protein |  | 1.50 | 1 |
| NCU05770 | catalase-2 |  | 1.50 | 1 |
| NCU09308 | glycoprotease |  | -1.50 | 1 |
| NCU06666 | Inositol |  | -1.55 | 2 |
| NCU02167 | Krev-1-like |  | -1.57 | 1 |
| NCU02174 | hypothetical protein |  | -1.60 | 1 |
| NCU06351 | phytase-1 |  | -1.62 | 1 |
| NCU04132 | hypothetical protein |  | -1.64 | 1 |
| NCU08005 | NADPH-adrenodoxin reductase Arh1 |  | -1.65 | 2 |
| NCU17088 | Aspartyl amino peptidase |  | -1.65 | 2 |
| NCU00399 | Cell wall protein PhiA |  | -1.79 | 2 |
| NCU01931 | hypothetical protein |  | -1.98 | 2 |
| NCU09909 | Urea active transporter |  | -2.18 | 1 |
| NCU02235 | Glycosyl hydrolase family 47-6 |  | -6.03 | 3 |

Gene expression values are expressed in log_2_ fold change between each condition.

**low- and high-Pi (**∆***pac-3 vs*** ∆***mus-52*)**

| **ID** | **Gene Product Name** | **low-Pi** | **high-Pi** | **Consensus** |
| --- | --- | --- | --- | --- |
| NCU07129 | amino-acid permease inda1 | 7.16 | 7.82 | 3 |
| NCU04197 | CipC protein | 6.62 | 8.44 | 1 |
| NCU08739 | Endothiapepsin | 5.34 | 5.27 | 1 |
| NCU00790 | High affinity potassium transporter-1 | 5.20 | 4.99 | 1 |
| NCU07083 | hypothetical protein | 4.19 | 3.37 | 1 |
| NCU06328 | hypothetical protein | 4.12 | 4.43 | 2 |
| NCU10387 | Dimethylaniline monooxygenase | 3.97 | 4.10 | 1 |
| NCU00282 | hypothetical protein | 3.89 | 4.34 | 1 |
| NCU03422 | hypothetical protein | 3.73 | 3.25 | 4 |
| NCU09305 | hypothetical protein | 3.70 | 3.06 | 1 |
| NCU04865 | polyketide synthase-3 | 3.37 | 3.42 | 2 |
| NCU05908 | hypothetical protein | 3.29 | 2.75 | 2 |
| NCU05105 | glucan endo-1,3-beta-glucosidase | 3.24 | 4.41 | 1 |
| NCU02877 | hypothetical protein | 3.14 | 3.95 | 1 |
| NCU07723 | Norsolorinic acid reductase | 3.08 | 2.26 | 1 |
| NCU09505 | hypothetical protein | 2.84 | 3.67 | 1 |
| NCU09724 | hypothetical protein | 2.76 | 3.74 | 1 |
| NCU02875 | hypothetical protein | 2.70 | 2.68 | 2 |
| NCU03240 | hypothetical protein | 2.67 | 5.05 | 1 |
| NCU06327 | benzoate 4-monooxygenase cytochrome P450 | 2.66 | 2.75 | 2 |
| NCU08418 | tripeptidyl-peptidase | 2.53 | 2.64 | 2 |
| NCU04046 | hypothetical protein | 2.27 | 2.98 | 4 |
| NCU05832 | hypothetical protein | 2.23 | 2.19 | 1 |
| NCU02213 | hypothetical protein | 2.18 | 2.94 | 1 |
| NCU08230 | hypothetical protein | 2.15 | 1.89 | 1 |
| NCU09772 | hypothetical protein | 2.13 | 3.33 | 2 |
| NCU06305 | Tartrate transporter | 2.13 | 2.32 | 1 |
| NCU11292 | hypothetical protein | 2.07 | 2.95 | 2 |
| NCU04866 | All development altered-6 | 2.00 | 2.15 | 1 |
| NCU09422 | hypothetical protein | 1.99 | 1.87 | 3 |
| NCU01881 | hypothetical protein | 1.87 | 2.03 | 1 |
| NCU07923 | hypothetical protein | 1.79 | 1.60 | 2 |
| NCU05395 | hypothetical protein | 1.75 | 1.66 | 1 |
| NCU09185 | hypothetical protein | 1.74 | 2.44 | 3 |
| NCU09182 | Stress responsive A/B barrel domain-containing protein | 1.73 | 2.51 | 3 |
| NCU04442 | GAL10 | 1.73 | 1.77 | 1 |
| NCU06239 | hypothetical protein | 1.64 | 1.57 | 1 |
| NCU08055 | b-ZIP transcription factor IDI4 | 1.60 | 2.45 | 2 |
| NCU01298 | hypothetical protein | 1.59 | 4.15 | 1 |
| NCU04276 | hypothetical protein | 1.58 | 5.48 | 2 |
| NCU08127 | Glycosyl hydrolase family 76-3 | 1.57 | 2.13 | 1 |
| NCU16992 | Mating factor a-1 | 1.55 | 1.57 | 1 |
| NCU06111 | GTPase Ras2p | 1.55 | 1.52 | 1 |
| NCU06772 | hypothetical protein | -1.51 | -1.90 | 2 |
| NCU04936 | UDP-glucose 6-dehydrogenase | -1.59 | -1.63 | 2 |
| NCU04569 | 5-oxoprolinase | -1.59 | -1.78 | 1 |
| NCU08895 | PNS1 | -1.79 | -1.87 | 4 |
| NCU09629 | hypothetical protein | -1.81 | 2.12 | 1 |
| NCU04292 | branched-chain-amino-acid aminotransferase | -1.85 | -1.62 | 1 |
| NCU04433 | cysteine-14 | -1.97 | -2.74 | 3 |
| NCU01781 | hypothetical protein | -2.05 | -2.16 | 3 |
| NCU00155 | C6 transcription factor | -2.10 | -1.86 | 1 |
| NCU03921 | Mitochondrial chaperone bcs1 | -2.12 | 2.15 | 1 |
| NCU06125 | hypothetical protein | -2.14 | -1.53 | 1 |
| NCU05805 | Serine hydroxymethyl transferase | -2.43 | -1.57 | 2 |
| NCU03639 | Lípase | -2.54 | -3.46 | 5 |
| NCU07748 | hypothetical protein | -2.62 | -4.94 | 1 |
| NCU01065 | Ammonium transporter MEP2 | -2.65 | -3.10 | 1 |
| NCU09935 | hypothetical protein | -2.72 | -2.86 | 2 |
| NCU04230 | acetate utilization-3 | -2.74 | -1.89 | 2 |
| NCU07953 | alternative oxidase-1 | -2.78 | -2.47 | 1 |
| NCU06061 | oxidoreductase | -2.82 | -2.34 | 2 |
| NCU07117 | ornithine-N5-oxygenase | -3.02 | -2.28 | 3 |
| NCU08439 | leptomycin B resistance protein pmd1 | -3.11 | -2.28 | 1 |
| NCU10038 | Glycerophosphoryl diesterphosphodiesterase | -3.17 | -4.04 | 3 |
| NCU04466 | Cyanamide hydratase | -3.30 | -2.63 | 2 |
| NCU02361 | formamidase | -3.35 | -3.55 | 1 |
| NCU07016 | hypothetical protein | -3.41 | -3.21 | 1 |
| NCU03153 | hypothetical protein | -3.42 | -3.40 | 2 |
| NCU07253 | 1,3-beta-glucanosyltransferase gel1 | -3.56 | -3.26 | 1 |
| NCU01066 | l-aminoacid oxidase | -3.60 | -4.48 | 4 |
| NCU06062 | Aerobactin siderophore biosynthesis protein iucB | -3.63 | -2.69 | 1 |
| NCU07966 | calcium-transporting ATPase 3 | -4.00 | -3.88 | 2 |
| NCU06063 | long-chain-fatty-acid-CoAligase | -4.26 | -3.38 | 1 |
| NCU08183 | hypothetical protein | -4.51 | -3.65 | 1 |
| NCU09271 | hypothetical protein | -4.57 | -4.85 | 2 |
| NCU02879 | zinc/iron transporter | -4.61 | -4.32 | 1 |
| NCU08325 | phosphorus-5 | -5.04 | -3.86 | 1 |
| NCU04452 | menadione-induced gene-3 | -5.18 | -5.77 | 2 |
| NCU02880 | hypothetical protein | -6.00 | -6.64 | 1 |
| NCU05046 | E1-E2 ATPase-1 | -6.77 | -5.72 | 2 |
| NCU07894 | Oligopeptide transporter 2 | -6.85 | -5.55 | 2 |
| NCU01064 | hypothetical protein | -8.84 | -5.77 | 3 |
| NCU09564 | phosphorus-4 | -9.74 | -7.06 | 3 |

Gene expression values are expressed in log_2_ fold change between each condition.
